# 3D Chromatin Architecture Provides Insights Into Leaf Trait Variation Among Pear Species

**DOI:** 10.1002/advs.202519321

**Published:** 2026-05-12

**Authors:** Yueyuan Liu, Chenhui Han, Cheng Xue, Manyi Sun, Bobo Song, Yongsong Xue, Guangyan Yang, Shaozhuo Xu, Wei Wei, Jiaming Li, Runze Wang, Jun Wu

**Affiliations:** ^1^ State Key Laboratory of Crop Genetics and Germplasm Enhancement, College of Horticulture Nanjing Agricultural University Nanjing Jiangsu China; ^2^ The Laboratory of Pear Germplasm Resources Innovative Utilization and Molecular Breeding, College of Horticulture Anhui Agricultural University Hefei Anhui China; ^3^ Zhongshan Biological Breeding Laboratory Nanjing Jiangsu China; ^4^ College of Horticulture Science and Engineering Shandong Agricultural University Tai'an Shandong China

**Keywords:** 3D genome, domestication and divergence, pear leaf, structural variation, whole‐genome duplication

## Abstract

Three‐dimensional (3D) chromatin architecture plays a fundamental role in eukaryotic gene regulations, its functional significance in perennial fruit trees remains poorly characterized despite extensive applications in crop genomics. Here, we developed high‐resolution (∼5 kb) Hi‐C maps of *Pyrus* and compared 3D genomic architecture of three representative pear species: the wild Asian pear ‘Duli’ (*P. betuleafolia*), the cultivated Asian pear ‘Dangshansuli’ (*P. bretschneideri*), and the cultivated European pear ‘Early red Doyene du Comice’ (*P. communis*). Approximately 78% of compartment and 73% of TAD‐like domains are conserved among the three species. Interspecific structural variation correlates with TAD‐like boundary repositioning, which is associated with altered expression levels of genes near these boundaries. The variations in TAD‐like boundaries among three species correlated with differentially expressed whole‐genome duplication (WGD) and single‐copy genes. Furthermore, integration of pear population data identified 234 domestication‐ and 3605 divergence‐associated genes near TAD‐like boundaries, which are enriched in cell development pathways. One candidate gene—*PyYABBY*, located in a different TAD‐like boundary between *P. bretschneideri* and *P. communis*, is confirmed to enhance leaf size. This study revealed 3D genomic divergence among three *Pyrus* species, demonstrating the functional and evolutionary roles of TAD‐like structures in genome organization, thereby providing insights for crop trait evolution research.

**Abbreviations**
3D: Three‐dimensional
ACR: Accessible Chromatin Regions
ATAC: Assay for Transposase‐Accessible Chromatin
DACRs: Differential enrichment of ACRs
DEG: Differentially expressed gene
FPKM: Fragments per kilobase per million mapped reads
GO: Gene Ontology
RNA‐seq: RNA sequencing
SVs: Structural variation
TAD: Topologically associating domain
TSS: Transcription start site
TTS: Transcription termination site
WGD: Whole Genome Duplication
WGS: Whole genome sequencing

## 1 Introduction

The three‐dimensional (3D) organization of chromatin in the genome strongly influences gene transcription, DNA replication and repair, and the regulation of several biological processes in eukaryotic organisms [1, 2, 3, 4]. Self‐interacting genomic units, which are topologically associating domains (TADs), can affect gene regulation [5]. TADs were characterized as local packing units demarcated by boundaries enriched with CTCF insulator binding sites and highly expressed genes, which are a hallmark of metazoan genomes [4, 6]. However, TADs are not a prominent feature in the genome of *Arabidopsis thaliana*, a phenomenon attributed to the absence of canonical insulator proteins in this and other plant species [7]. Therefore, the chromatin structures detected by Hi‐C in plants are typically termed ‘TAD‐like’. The utilization of 3D genomics can aid in mapping the spatial organization of chromatin in plants, shedding light on its potential role in governing gene transcription [8]. In *Arabidopsis*, a high‐resolution Hi‐C map at the gene level was constructed, revealing a positive correlation between gene expression and gene loops [9]. In rice, exploring promoter‐promoter interactions identified frequent co‐transcription of interacting genes, as illustrated in a promoter‐promoter interaction map involving RNA polymerase II (RNAPII) and H3K4me3 [10]. Chromatin interaction maps in these species are instrumental for mapping associations between genomic loci, including genes and candidate regulatory elements.

Comparative research has indicated that chromatin structural alterations play a significant role in driving cell differentiation, embryo development, and genome evolution [11, 12, 13, 14, 15]. By comparing the subgenomes in tetraploid cotton plants to their present diploid ancestor, genome allopolyploidization likely contributed to A/B compartment switching, and TAD restructuring in both subgenomes was found. During polyploidization, TAD boundaries may arise preferentially in open chromatin regions, coinciding with chromatin modifications [16]. By constructing high‐resolution (2 kb) Hi‐C maps of cultivated soybean (*Glycine max*), wild soybean (*Glycine soja*), and common bean (*Phaseolus vulgaris*), polyploidization in soybean was observed, and may have resulted in architectural alterations of TADs, and subsequent diploidization led to chromatin topology changes near chromosome rearrangement sites [17]. However, research on the 3D genome architecture and interspecific diversity in 3D genome structure among perennial fruit trees is limited.

Leaves are essential organs for plant growth and development [18]. A diverse range of leaf characteristics, including sizes and shapes, facilitates the adaptation of higher plants to various environments and habitats [19, 20]. Since the leaves serve as the principal locus for photosynthesis, alterations in their shape may influence photosynthesis efficiency, affecting the amount of sugar (BRIX) and fruit yield. In tomatoes, photosynthesis contributes significantly to plant biomass and fruit sugar content but negatively affects yield. On the contrary, the shape of the leaves, especially the round ones, had a strong positive effect on both fruit sugar content and yield. The rounder the leaf, the better the quality of the fruit [21]. Consequently, examining leaf shape and size across species is essential for comprehending variations in fruit qualities. In *Brassica napus*, 3D chromatin compartments are associated with genetic variation among parents. Genes associated with hormones and the cell cycle are increasingly up‐regulated due to alterations in the 3D genome, promoting leaf growth [22]. In *Populus*, a comprehensive analysis of Hi‐C‐seq, ATAC‐seq, methylation, and gene expression demonstrated that longer 3D genome interactions, hypomethylation, and an open chromatin state enhance the expression of IAA‐related genes (e.g., *PIN‐FORMED1* and *ANGUSTIFOLIA3*) and facilitate the development of broad leaves. Conversely, narrow leaves were linked to dense heterochromatin, hypermethylation, and the upregulation of abscisic acid pathway genes (e.g., *Pyrabactin Resistance1‐like10*) [23]. However, interspecific variations in the 3D genomes of perennial fruit tree leaves have not been investigated.

The *Pyrus* genus is prolific within the *Rosaceae* family, with a minimum of 22 distinct species and over 5,000 documented or preserved accessions worldwide [24, 25]. Based on geographical distribution, pears are classified into two major cultivated groups: Asian pears and European pears. The first complete genome of the ‘Dangshansuli’ Chinese white pear, often referred to as Asian pear (*P. bretschneideri Rehd*.), was compiled in 2013, revealing that 53% of the pear genome comprises repetitive sequences [26]. *P. betuleafolia* is an ancestral species linked to both Oriental and Occidental pear [27, 28]. It exhibits a robust root system, vigorous growth, and resistance to diverse environmental and biological stressors [29, 30]. The European pear, *P. communis L*., is an economically significant species widely cultivated in Western countries, exhibiting various phenotypic and fruit quality traits that differ from those of the Asian pear, including variations in fruit shape, taste, lignin content, and aroma [31]. According to the phylogenetic tree, ‘Dangshansuli’ (*P. bretschneideri*) is classified in Asian pear Group I, and cultivated European pear ‘Early red Doyene du Comice’ (*P. communis*) belongs to European pear Group II [31]. However, no investigations have been undertaken to compare the 3D chromatin structures of different pear species. The variations in leaf shape and size among species have not been examined by viewing the 3D genome of leaves across species.

In this study, we constructed Hi‐C maps of the ‘Dangshansuli’ pear, comparing the 3D architecture of wild Asian, cultivated Asian, and cultivated European pear species to clarify variations in leaf traits using Hi‐C, ATAC‐seq, PacBio HiFi long reads, and RNA‐seq data. We investigated interspecific variations in 3D chromatin structure concerning interspecific SVs, expression levels of duplicated genes, and population domestication and divergence, highlighting the evolution and function of TAD‐like domains and TAD‐like boundaries. In addition, we combined the phenotypic variations in the leaves of the three species and discovered that genes located near the boundary of the differential TAD‐like boundaries were enriched in cell fate, jasmonic acid, and other hormonal pathways associated with leaf development and resistance. *PyYABBY* was identified as a candidate WGD gene and was positioned in a genomic region annotated as a TAD‐like boundary; independent functional assays validated its role in promoting leaf development and increasing leaf size. The results shed light on the unique 3D chromatin architectures in three *Pyrus* species and underscore the functional and evolutionary importance of TAD‐like domains in genome organization.

## 2 Results

### 2.1 Genome Assembly of ‘Dangshansuli’ Pear

The genome assembly of ‘Dangshansuli’ pear was constructed by integrating PacBio HiFi reads (24.74 Gb, ∼48× genome coverage), Hi‐C data (51.86 Gb, ∼101× genome coverage) [32]. After assembling, we obtained a preliminary draft assembly consisting of 352 contigs with a total size of 519.45 Mb. The contig N50 was 29.67 Mb (Table 1). The Hi‐C data were used to construct chromosome‐level scaffolds. The completeness of the genome assembly was evaluated using BUSCO, and 99.63% (1,608/1,614) of the core eukaryotic genes were detected in the assembly. Using the EDTA pipeline [33], we annotated transposable elements (TEs) in the ‘Dangshansuli’ hifi genome, revealing that 48.2% of the assembly consists of TEs, with long terminal repeats (LTR) retrotransposons being the most abundant class (Table S1). Based on the annotation of LTRs, the calculated LAI score was 20.51. As this value exceeds the threshold of 20, it meets the established criterion for a gold‐quality genome assembly [34].

**TABLE 1:** Statistics of genome assembly and annotation of ‘Dangshansuli’.

Assembly	‘Dangshansuli’
Total length (Mb)	519.45
Contig number	17
Contig length (Mb)	519.45
Largest contig (Mb)	41.23
Contig N50 (Mb)	296.74
GC content of the genome (%)	37.93
BUSCO completeness of genome assembly(%)	99.63
Number of predicted protein‐coding genes	41,048
Masked repeat sequence length (Mbp)	241.69

A total of 41,048 protein‐coding genes were predicted in the ‘Dangshansuli’genome. Functional annotation of the 40,064 predicted protein‐coding genes was performed against six public databases. The numbers of genes annotated by the eggNOG, KEGG, Pfam, Swiss‐Prot, Gene Ontology (GO), and NR databases were 37,687; 17,150; 32,563; 27,135; 18,717; and 39,237, respectively (Table S2). These results showed that we have successfully obtained a genome reference of ‘Dangshansuli’ pear, with high continuity completeness, and base accuracy.

### 2.2 3D Genomic Characterization of Reference ‘Dangshansuli’ Pear

To unveil the 3D chromatin structure of the ‘Dangshansuli’ pear, we conducted Hi‐C experiments with two biological replicates. A correlation coefficient of 0.91 was observed between the two replicates at 250 kb resolution (Figure S1). APA analysis was also conducted and the average P2LL (Peak‐to‐Local Loading) value for the identified loops exceeded 4 at resolutions of 25 kb (P2LL = 4.583), 10 kb (P2LL = 4.343), and 5 kb (P2LL = 7.895), indicating the presence of strong, highly significant chromatin interactions and reflecting high‐quality data and reliable results (Figure S2). Subsequently, the two replicates were merged, resulting in 674 million sequencing reads used to construct Hi‐C maps. From this, 281 million valid interaction pairs were derived and utilized in constructing Hi‐C maps (Table S3), enabling the identification of compartment A/B, TAD‐like domains, and loops.

**FIGURE 1:**
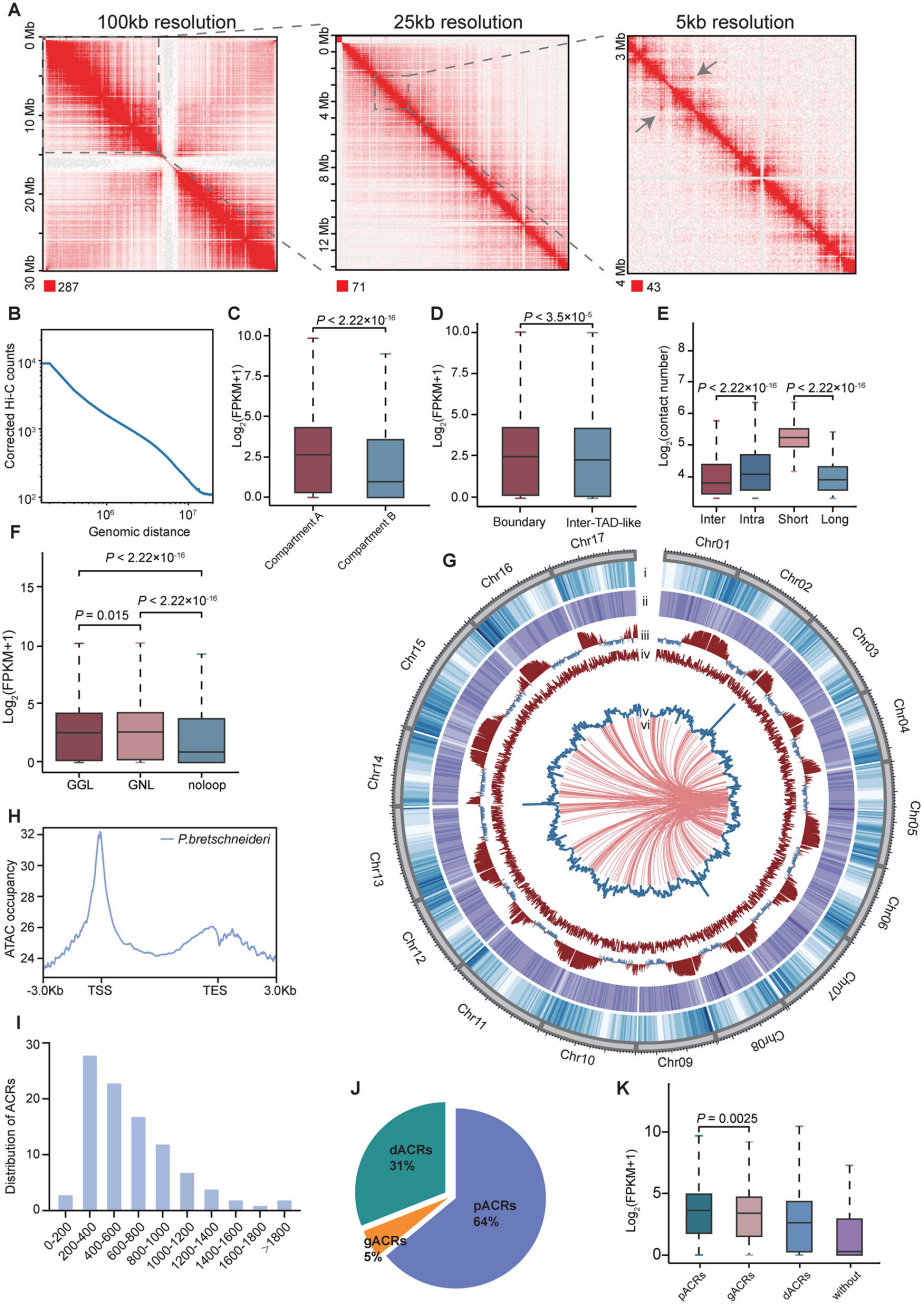
Global 3D genome organization of reference ‘Dangshansuli’ pear. (A) Hi‐C maps of Chr03 in ‘Dangshansuli’ pear with different resolutions (100, 25 and 5 kb). The loop structures are indicated by the gray arrows. (B) Genomic distance vs. contact counts plot using Hi‐C matrices in ‘Dangshansuli’ pear. (C) Expression level of genes in compartment A (*n* = 28,963) and compartment B (*n* = 8963). (D) Expression level of genes in TAD‐like boundary (*n* = 15,227) and TAD inter region (*n* = 21,958). (E) Contact numbers of inter‐ (*n* = 93,649), intra‐chromosomal (*n* = 289,737), short (≤ 2 Mb) (56,653) and long (> 2 Mb) (233,083) loops in 'dangshansuli' pear. (F) Expression level of genes in gene‐gene loops (12,382), gene‐none loops (*n* = 18,488), and without loops (*n* = 21,222). (G) Circle plot of gene density (i), TE density (ii), compartments A/B (iii), TAD‐like boundary scores (iv), ATAC peaks (v), and loops (vi) in the pear whole genome. (H) ATAC intensity in the ‘Dangshansuli’ genome. (I) Length distribution of ATAC‐seq peaks (bp) in the ‘Dangshansuli’ genome. (J) ACRs are subdivided based on their distribution pattern around genes in the ‘Dangshansuli’ genome. (K) Expression levels of pACR (*n* = 16,110), gACR (*n* = 1,549), and dACR (*n* = 5,766), and absence of gene peaks. The *P*‐values in C, D, E, K were based on the two‐tailed Wilcoxon's rank‐sum tests. The *p‐*values in Figure F were adjusted for multiple comparisons using the Benjamini‐Hochberg procedure (FDR) following Wilcoxon's rank‐sum tests. In C, D, E, F and K, each box represents the center line (median), the box limits (first and third quartiles) and the whiskers (1.5× the IQR).

The matrix resolution, as described in a previous study, refers to the level of resolution at which 80% of loci have 1000 or more contacts with any other locus [35]. In our study, we constructed high‐quality Hi‐C maps at 100 kb, 25 kb, and 5 kb resolution. Displaying the chromatin interaction maps in Figure 1A, the results indicated a rapid decrease in the frequency of intra‐chromosomal interaction with linear distance (Figure 1B). Hi‐C data highlighted a prevalence of intra‐chromosomal interactions over inter‐chromosomal interactions in pear (Table S3). A total of 10039 compartment A/B bins were identified based on the Hi‐C matrix generated by Juicer at a 50 Kb resolution, with 59% of the genome belonging to compartment A. The expression level of genes in compartment A showed significantly higher than that observed in compartment B (Figure 1C, Wilcoxon rank‐sum test). Notably, compartment B exhibited a significantly higher TE density than compartment A (Figure S3A).

Furthermore, 6,952 TAD‐like domains (Table S4) and 7,000 TAD‐like boundaries were discerned using a 3D contact matrix at a 25 kb resolution, revealing that genes located in TAD‐like boundaries exhibited higher expression levels compared to those in TAD‐like interior region (Figure 1D, Wilcoxon rank‐sum test). Additionally, TEs were more enriched in inter‐TAD‐like regions compared to TAD‐like boundaries (Figure S3B). Among TEs localized at TAD‐like boundaries, LTR/known elements predominated, followed by DNA/DTM elements (Figure S3C).

At a 5 kb resolution, we identified 289,737 intra‐chromosomal loops and 93,649 inter‐chromosomal loops (contact number > 10, *Pp* < 0.05). The contact level of intra‐chromosomal loops was higher than that of inter‐chromosomal loops (Figure 1E, Wilcoxon rank‐sum test). The contact level of short loops was higher than that of long loops (Figure 1E, Wilcoxon rank‐sum test). Approximately 5.5% (16,036/289,737) of intra‐chromosomal loops had genes at both anchors, which were termed gene‐gene loops (GGLs), and 32.9% (95,248/289,737) of intra‐chromosomal loops had genes at one anchor. A total of 12,460 genes were connected by GGLs, and 18,576 genes were connected by gene‐non‐gene loops (GNL); the expression of genes linked by GGL and GNL was significantly higher than that of genes with no loops (Figure 1F, Wilcoxon rank‐sum test).

ATAC‐seq was conducted to understand the impact of enhancer‐like elements on the expression of genes in TAD boundaries (Figure 1H). We identified 33,658 accessible chromatin regions (ACRs) in the ‘Dangshansuli’ genome. The length of ACRs mainly fell between 200 and 1200 bp, and most were within 200–400 bp (Figure 1I). These regions have been classified into gene body (g), promoter (p), and distal (d) ACRs based on their location concerning genes. The prevalence of pACRs in the pear genome was highest (Figure 1J) relative to *Arabidopsis*, *Medicago*, and rice, but these findings were inconsistent with tomato and wheat [36, 37]. Like other crops, pACRs, gACRs, and dACRs were strongly correlated with transcriptional activity, with pACRs being associated with the highest expression levels (Figure 1K, Wilcoxon rank‐sum test). Compartment A exhibits a significantly higher density of accessible chromatin regions (ACRs) than compartment B (Figure S4).

### 2.3 Compartment A/B and TAD Alterations Among Pear Species

**FIGURE 2:**
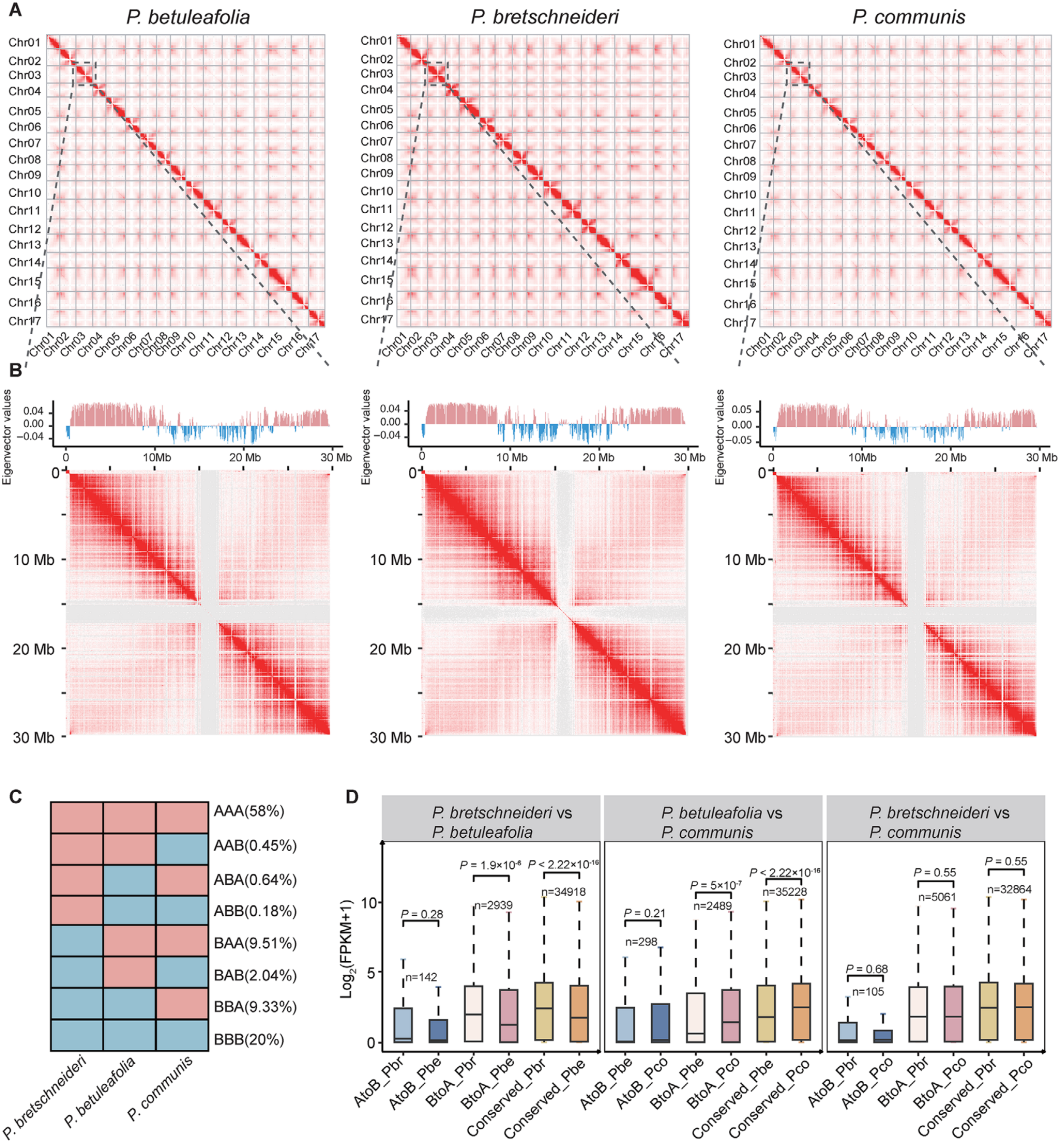
Hi‐C maps and compartment A/B comparison in *P. bretschneideri*, *P. betuleafolia*, and *P. communis*. (A) Hi‐C maps of the whole genome of *P. bretschneideri*, *P. betuleafolia*, and *P. communis* (2.5 Mb resolution). (B) The Eigenvector value of A/B chromatin compartments of Chr03 (50 kb resolution). (C) Number of dynamic A/B chromatin compartment bins. (D) The gene expression levels in conserved and switching compartments (A to B and B to A) were compared between three combinations: *P. bretschneideri* vs. *P. betuleafolia*, *P. betuleafolia* vs. *P. communis*, and *P. bretschneideri* vs. *P. communis* (Abbreviations: Pbr: *P. bretschneideri*; Pbe: *P. betuleafolia*; Pco: *P. communis*). The number (*n*) displayed below each *P‐*value indicates the number of genes in each group.

The whole genome Hi‐C maps (2.5 Mb resolution) of the wild Asian pear ‘Duli’ (*P. betuleafolia*), the cultivated Asian pear ‘Dangshansuli’ (*P. bretschneideri*), and the cultivated European pear ‘Early red Doyene du Comice’ (*P. communis*) were shown in Figure 2A. To compare the 3D chromatin structure of the three different pear species, their leaves were collected for Hi‐C library construction and Hi‐C sequencing, which returned 674 million sequencing reads for constructing Hi‐C maps (Table S3). The newly assemble the ‘Dangshansuli’ pear genome was used as the reference genome. Hi‐C reads were mapped to the reference genome and revealed 203–281 million valid contact reads.

To characterize variations between the 3D genome structures of the three pear species, we categorized the chromatin of each species, using a 50 kb Hi‐C matrix, into A and B compartments representing active and inactive chromatin regions, respectively, then compared the A/B compartments and TAD compositions. Both *P. betuleafolia* and *P. communis* had the same number of A/B compartments as *P. bretschneideri*, respectively, and more genomic regions occurred in compartment A (Figure S5A). When comparing compartment A/B (50 kb resolution) among the three species, we found that 78% of compartment A/B was conserved at the global level (Figure 2B). Among all three species, the *P. communis* exhibited the largest proportion of dynamic switching regions (Figure 2B). RNA‐seq analysis was conducted using RNA isolated from leaves (3 replicates per plant) of the ‘Dangshansuli’, ‘Duli’ and ‘Early red Doyene du Comice’ pears, and the DEGs are shown in Figure S6. To address the effect of 3D genomic structural variation on gene expression, we first investigated the relationship between gene expression and the A/B compartments, then incorporated transcriptomic data to determine the expression levels of genes in the conserved and variable regions of each combination. The FPKM values of genes in the conserved and switched compartments of the A/B region showed significant differences between the wild and the cultivated species (Figure 2D, Wilcoxon rank‐sum test). In contrast, no significant differences were found between the two cultivated species (Figure 2D, Wilcoxon rank‐sum test).

**FIGURE 3:**
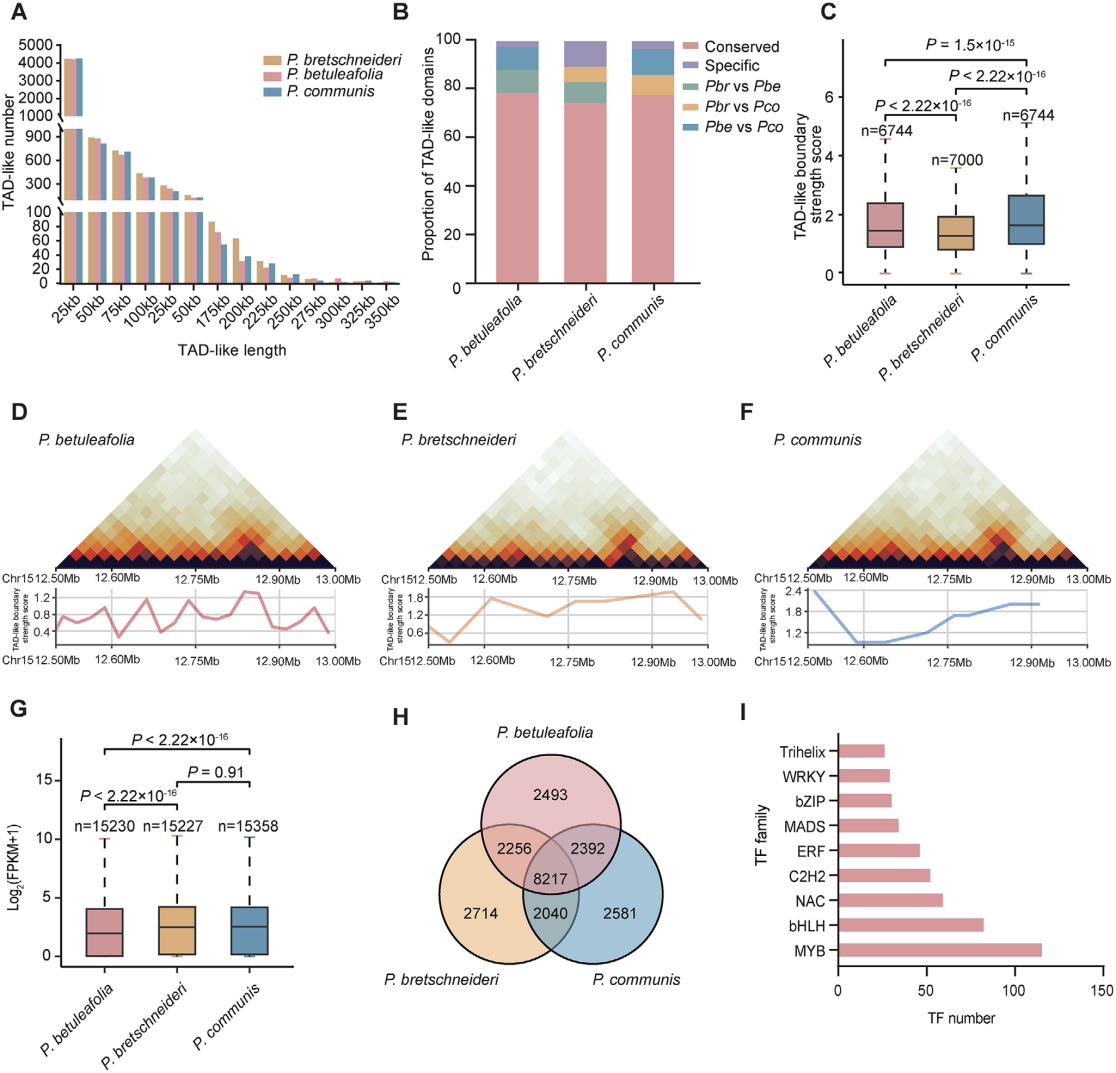
Global TAD‐like organization in *P. bretschneideri*, *P. betuleafolia*, and *P. communis*. (A) The size of TAD‐like domains in *P. bretschneideri*, *P. betuleafolia* and *P. communis*. (B) The conserved TAD‐like domains among *P. bretschneideri*, *P. betuleafolia*, and *P. communis* (‘Conserved’ refers to TAD‐like domains conserved across the three pear species; ‘Specific’ denotes species‐specific TAD‐like domains. Abbreviations: Pbr: *P. bretschneideri*; Pbe: *P. betuleafolia*; Pco: *P. communis*). (C) The TAD‐like boundary scores in *P. bretschneideri* (*n* = 7,000), *P. betuleafolia* (*n* = 6,744), and *P. communis* (*n* = 6744). (D–F) Heat map (25 kb resolution) of TAD‐like domains on chromosome 5: 12.5‐13Mb among *P. bretschneideri*, *P. betuleafolia*, and *P. communis*. (G) The expression level of genes in TAD‐like boundaries among *P. bretschneideri* (*n* = 15,227), *P. betuleafolia* (*n* = 15,230), and *P. communis* (*n* = 15,358). (H) The overlap genes in TAD‐like boundaries among *P. bretschneideri*, *P. betuleafolia*, and *P. communis*. (I) The number of transcription factors (TFs) in conserved TAD‐like boundaries among *P. bretschneideri*, *P. betuleafolia*, and *P. communis*.

The reorganization of TADs could result in alterations of chromatin interaction patterns at TAD boundaries, potentially impacting gene expression [13, 38]. Significant variations in TAD organization were observed between the three species. Using 25 kb resolution matrices, 6,952 TAD‐like domains in *P. bretschneideri*, 6,679 in *P. betuleafolia*, and 6,671 in *P. communis* were identified (Figure 3A). 5,106 TAD‐like domains were conserved among the three species. Further analysis showed that 748, 217, and 277 TAD‐like domains were specific among *P. bretschneideri*, *P. betuleafolia*, and *P. communis*, respectively (Figure 3B). The TAD‐like boundaries strength score among the three species differed significantly (Figure 3C, Wilcoxon rank‐sum test). The TAD‐like domains heatmaps and corresponding TAD‐like boundary strength scores of three species were displayed in Figure 3D–F.

Upon examining the number of genes occurring within TAD‐like boundaries, we found that those in *P. bretschneideri* and *P. communis* exhibited much greater differences in expression levels compared to the wild *P. betuleafolia*, whereas *P. bretschneideri* and *P. communis* did not show any significant expression differences between each other (Figure 3G, Wilcoxon rank‐sum test). These findings suggested that the variations between the wild and cultivated species are more significant than the differences observed between the cultivated species. When we compared the genes in TAD‐like boundaries among three species, 8,217 genes were conserved (Figure 3H). By integrating the genes conserved among three species in the TAD‐like boundary, we next attempted to identify associated transcription factors (TFs) and found mostly MYB, bHLH, NAC, C2H2, and ERF TF families (Figure 3I).

To evaluate the genome‐wide relationship between TAD‐like boundary conservation and gene expression divergence, we systematically compared expression levels between conserved and non‐conserved TAD boundaries across species. Expression divergence was quantified using |Log_2_(FPKM1 + 0.0001) / (FPKM2 + 0.0001)|, with genes satisfying |Log_2_(FPKM1 + 0.0001) / (FPKM2 + 0.0001)| ≥ 1 considered differentially expressed at synergistic boundaries. Across all pairwise comparisons, a substantial proportion of genes in non‐conserved TAD regions exhibited significant expression changes: i) Between *P. bretschneideri* and *P. betuleafolia*: 45.33% (3,436/7,579); ii) Between *P. bretschneideri* and *P. communis*: 38.66% (2,107 / 5,450); iii) Between *P. betuleafolia* and *P. communis*: 50.26% (2,350/4,675) (Figure S7).

### 2.4 SVs Among Pear Species May be Related to TAD‐Like Boundary Alterations

**FIGURE 4:**
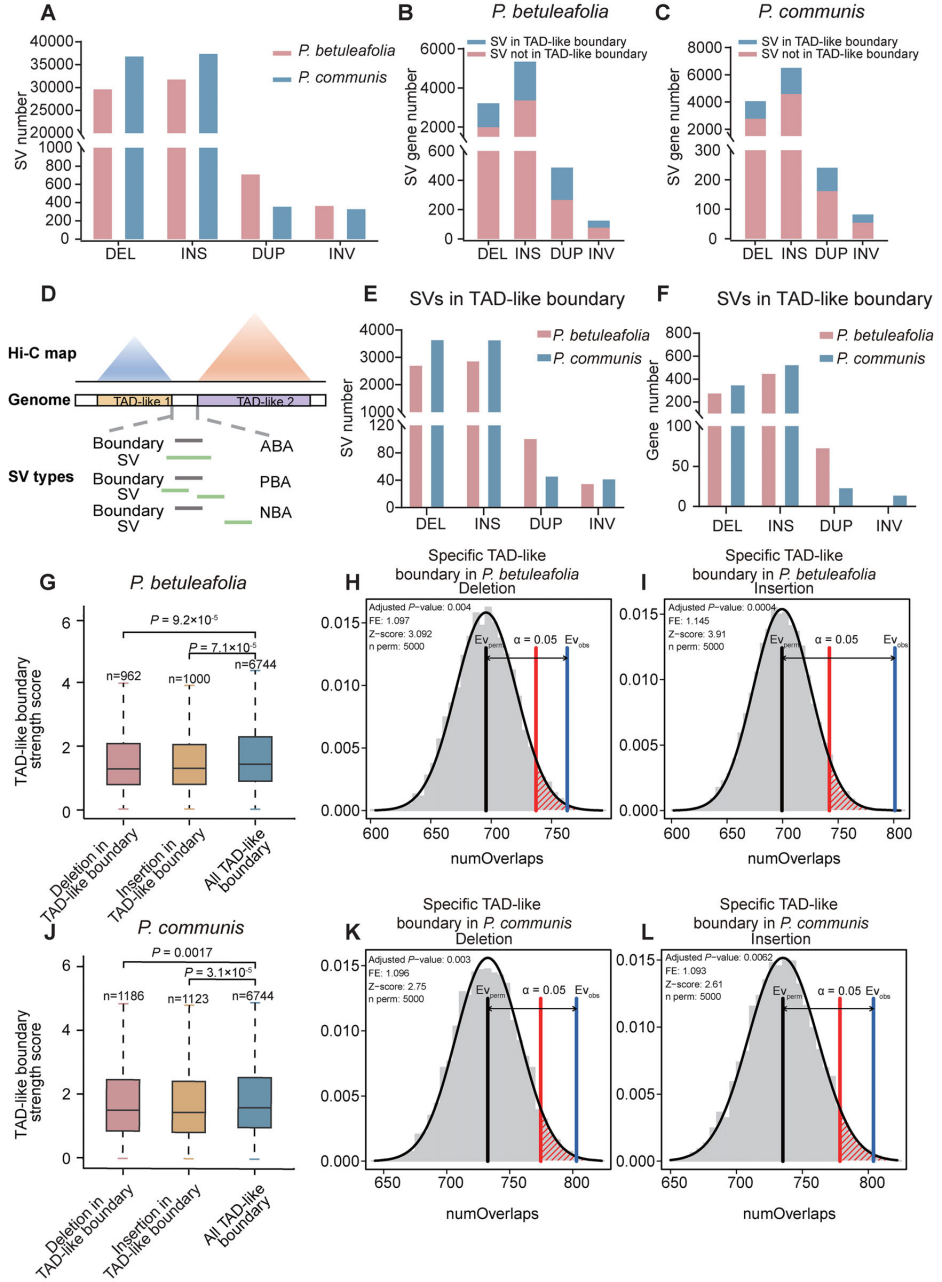
Distribution of SVs in TAD‐like boundaries among *P. bretschneideri*, *P. betuleafolia*, and *P. communis*. (A) The number of different types of SVs (DEL: deletion; INS: insertions; DUP: duplications; INV: inversions) in *P. betuleafolia* and *P. communis*. (B, C) The number of genes with SVs in *P. betuleafolia* and *P. communis*. The blue bars represent the number of genes within SVs at TAD‐like boundaries, while the pink bars represent the number of genes without SVs at TAD‐like boundaries. (D) Number of different SV types within TAD‐like boundaries. (E) The number of different types of PBA‐SVs in *P. betuleafolia* and *P. communis*. (F) The number of genes with PBA‐SVs in *P. betuleafolia* and *P. communis*. (G,J) TAD‐like boundary scores comparisons between SV‐associated TAD‐like boundaries and all TAD‐like boundaries in *P. betuleafolia* (G) and *P. communis*. (J) Number (*n*) below each *p‐*value indicates the number of TAD‐like boundaries in each group. (H, I, K, L) Expected (Evperm) and observed (Evobs) overlaps between SVs and specific TAD‐like boundaries, showing that observed overlaps exceed expectations. Grey indicates the distribution from randomized regions, blue the evaluation of the original region set, and red the significance threshold.

Previous studies have shown that long‐read sequencing depth of 15× is appropriate for identifying SVs in pear genomes [39]. The contributions of SVs to 3D genomic structural variation in perennial species have yet to be elucidated. Here, we characterized the landscape of SVs in the whole genome using PacBio‐HiFi long reads and investigated their potential associations. SVs of *P. betuleafolia* and *P. communis* species were identified using PacBio HiFi long reads with ‘Dangshansuli’ pear as the reference genome. More insertions and deletions were occurred in European cultivar *P. communis* than in Asian wild *P. betuleafolia* (Figure 4A), and a greater number of genes with deletions and insertions were in *P. communis* (Figure 4B,C). However, more duplications and inversions were in Asian wild *P. betuleafolia*, and the associated genes in *P. betuleafolia* were more than those in *P. communis*.

We then divided SVs into three categories according to studies done in soybean [40]: absolute boundary‐affecting (ABA) SVs were defined as SVs spanning the whole length of one boundary, partial boundary‐affecting (PBA) SVs were defined as SVs only spanning part of the length of one boundary, and non‐boundary‐affecting (NBA) SVs were defined as SVs within TAD‐like domains (Figure 4D). Most SVs belong to the NBA type, while ABA types were the lowest in number (Table S5). This result was consistent with similar findings in soybean and cotton [40, 41].

Next, we investigated the association between PBA SVs and alterations in TAD‐like boundaries. In TAD‐like boundaries, more PBVs and genes with SVs in *P. communis* (Figure 4E,F). We further compared strength scores between TAD‐like boundaries within deletions/insertions and typical TAD‐like boundaries in *P. betuleafolia* and *P. communis*. Notably, TAD‐like boundaries with SVs (deletions and insertions) exhibited significantly lower strength scores than all TAD‐like boundaries (Figure 4G,J, Wilcoxon rank‐sum test). What's more, we incorporated background genomic features (gene and TE density) and compared TAD‐like boundary strength scores. We defined regions as gene‐rich (top 20% of genome‐wide gene density, ≥80th percentile), gene‐poor (bottom 20%, ≤20th percentile), and intermediate (20%–80%). Regions were classified as TE‐rich (top 20%, ≥80th percentile), TE‐poor (bottom 20%, ≤20th percentile), and intermediate (20%–80%). When gene/TE density was within the 20%–80% range, TAD‐like boundary strength scores were consistent with the genome‐wide trends, whereas regions with extremely high or low gene/TE density exhibited deviations (Figure S8).

To investigate whether SVs are enriched at specific TAD‐like boundary regions, we performed permutation tests using the regioneR package [42, 43]. Specifically, for deletions and insertions, we conducted 5,000 permutation tests on 10,000 randomly sampled subsets; for duplications and inversions, 300 permutation tests were performed on 300 randomly sampled subsets. These tests evaluated whether the observed overlaps between SVs and their specific TAD‐like boundaries occurred at a frequency significantly greater than expected by chance. The results revealed significant enrichment of both deletions and insertions at non‐conserved TAD‐like boundaries in *P. betuleafolia* (deletions: *p* = 0.0012; insertions: *p* = 0.0092) (Figure 4H,I) and *P. communis* (deletions: *p* = 0.0008; insertions: *p* = 0.0006) (Figure 4K,L). In contrast, neither duplications nor inversions showed significant enrichment at these regions (Figure S9).

**FIGURE 5:**
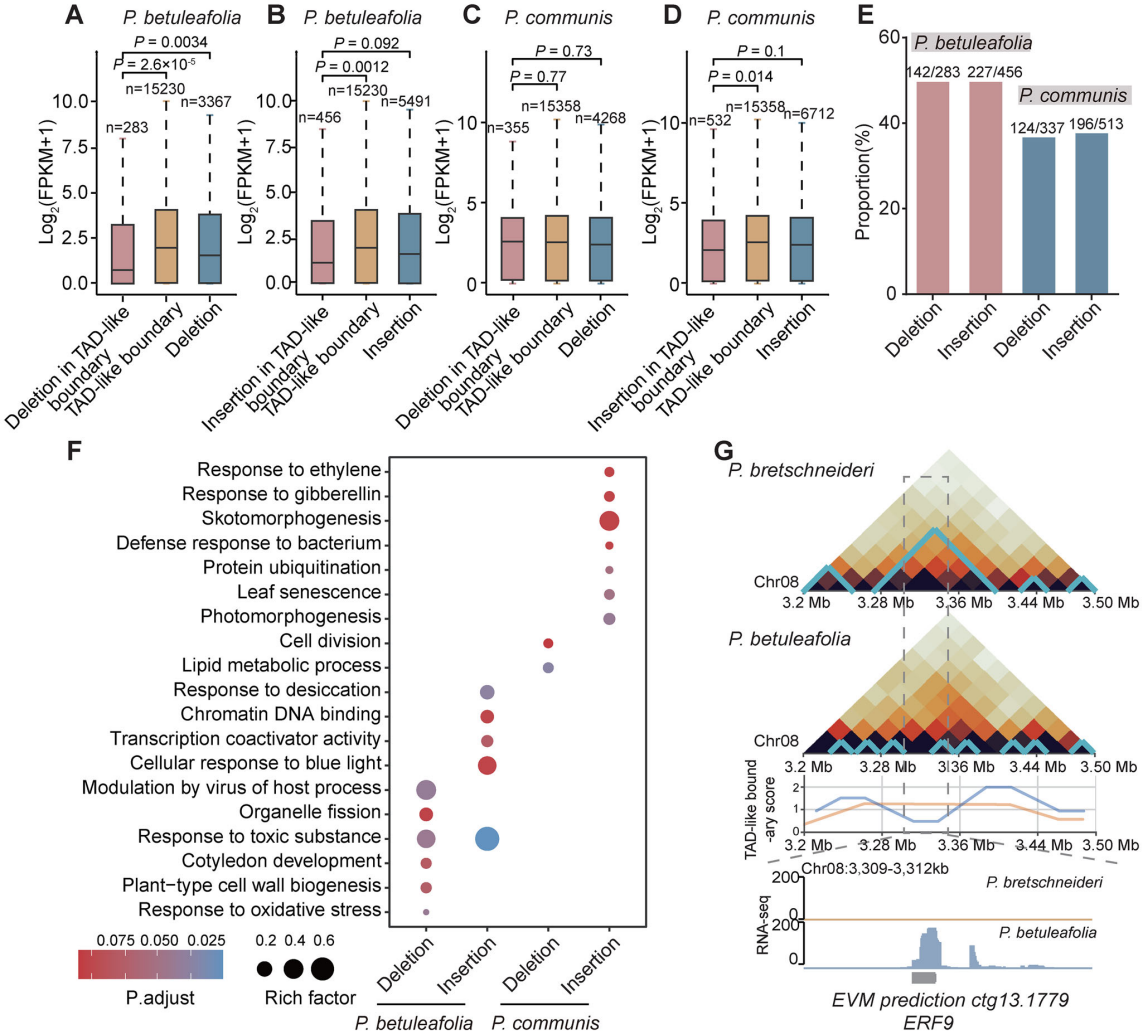
Expression patterns of genes associated with SVs at TAD‐like boundaries. (A, B) Comparison of expression levels of genes with SVs present at the TAD‐like boundaries, all genes at the TAD‐like boundaries, and all genes within SVs in *P. betuleafolia*. (C, D) Comparison of expression levels of genes with SV present at the TAD‐like boundaries, all genes at the TAD‐like boundaries, and all genes within SVs in *P. communis*. (E) The proportion of genes with expression differences among those harboring SVs at TAD‐like boundaries across different species. The proportion of differentially expressed genes was calculated as the number of genes with |Log_2_(FPKM1 + 0.0001) / (FPKM2 + 0.0001)| ≥ 1 divided by the total number of genes harboring SVs at specific TAD‐like boundaries. (F) GO pathway analysis of genes containing PBA‐SVs occurring within TAD‐like boundaries in *P. betuleafolia* and *P. communis*. (G) An example of one DEG (*ERF9*) containing PBA SVs in *P. bretschneideri* and *P. betuleafolia* is the TAD‐like heatmap (25 kb resolution) and RNA signals shown on the bottom. In A, B, C and D, the number (*n*) displayed below each *P‐*value indicated the number of genes in each sample.

Furthermore, we investigated differential expression level changes in genes in SVs at TAD‐like boundaries relative to all genes located in TAD‐like boundaries. Gene expression patterns across three groups were as follows: 1) genes within SVs in TAD‐like boundaries, 2) all genes in TAD‐like boundaries, and 3) all genes within SVs. In *P. betuleafolia*, genes with deletions at TAD‐like boundaries showed significantly low expression level compared to both general TAD‐like boundary genes and all genes within deletions (Figure 5A, Wilcoxon rank‐sum test). For insertions, boundary genes differed significantly from general TAD‐like boundary genes but not from all genes within insertions (Figure 5B, Wilcoxon rank‐sum test). In *P. communis*, only genes with insertions at TAD‐like boundaries displayed significant expression divergence from all genes in the TAD‐like boundary (Figure 5C,D, Wilcoxon rank‐sum test). We also performed a comprehensive sensitivity analysis by re‐identifying TAD‐like domains under multiple conditions, including the use of two biological replicates and different step sizes. When step size was held constant across replicates, our conclusions were consistent: 1) The TAD conservation rates for two biological replicates across three pear species are all above 80% (Figure S10); 2) correlation coefficients for TAD boundaries in both repetitions exceeding 0.8 (Figure S11); 3) TAD‐like boundary strength scores at boundaries harboring SVs were significantly lower than those of genome‐wide TAD‐like boundaries; and 4) at species‐specific TAD boundaries, genes with SVs exhibited significantly lower expression than the overall gene set at those boundaries (Figures S12 and S13).

We also controlled for background genomic features (gene and TE density) and compared the expression levels of SV‐overlapping genes at TAD‐like boundaries versus all boundary‐associated genes. Analysis demonstrated that across the moderate gene/TE density range (20%–80%), trends in boundary strength and associated gene expression aligned with genome‐wide patterns, whereas marked deviations were observed in regions of extreme gene/TE density (Figures S14 and S15).

We next quantified genes showing coordinated changes in TAD‐like structure and expression when SVs occur at TAD‐like boundaries. Between *P. bretschneideri* and *P. betuleafolia*, among 2,950 genes at *P. betuleafolia* specific boundaries, 244 harbored deletions, of which 126 (51.64%) showed coordinated TAD‐like and expression changes; among 422 genes with insertions, 211 (50.40%) showed coordinated changes (Figure 5E). Between *P. bretschneideri* and *P. communis*, among 3,082 genes at *P. communis* specific boundaries, 337 had deletions, of which 124 (36.80%) showed coordinated changes; among 513 genes with insertions, 196 (38.21%) showed coordinated changes (Figure 5E). These genome‐wide counts confirmed that SV‐induced TAD boundary alterations are frequently associated with gene expression changes beyond isolated examples.

After annotating these SVs, genes identified within regions of PBA SVs and in proximity to TAD‐like boundaries were subjected to GO analysis. The results showed that the ‘Response to toxic substance’, ‘Response to desiccation’, and ‘Cell division’ categories were enriched (Figure 5F). We observed that SVs in *P. betuleafolia* were associated with a reorganization of TAD‐like boundaries. The 2,619 bp deletion near the 3.3 M locus on chromosome 8 was associated with a shift in the TAD‐like boundary and a change in TAD‐like size between *P. bretschneideri* and *P. betuleafolia* (Figure 5G; Figure S16A,B). The reduction of these boundaries is accompanied by differential expression of three genes, including the significant transcription factor ERF9, which shows higher expression in *P. betuleafolia* than in *P. bretschneideri* (Figure S16C). The *Arabidopsis thaliana* AP2/ERF (APETALA2/ethylene response factor) family comprises plant‐specific transcription factors with a conserved DNA‐binding domain that regulate jasmonic acid (JA)‐ and ethylene‐mediated defense responses [44, 45]. We hypothesized that SVs are linked to TAD‐like organization in *P. betuleafolia* and the expression level of important genes within these domains, which are associated with divergent agronomic traits in pear species.

### 2.5 TAD‐Like Variation Linked to WGD Gene Expression Between Wild and Cultivated Pear Species

**FIGURE 6:**
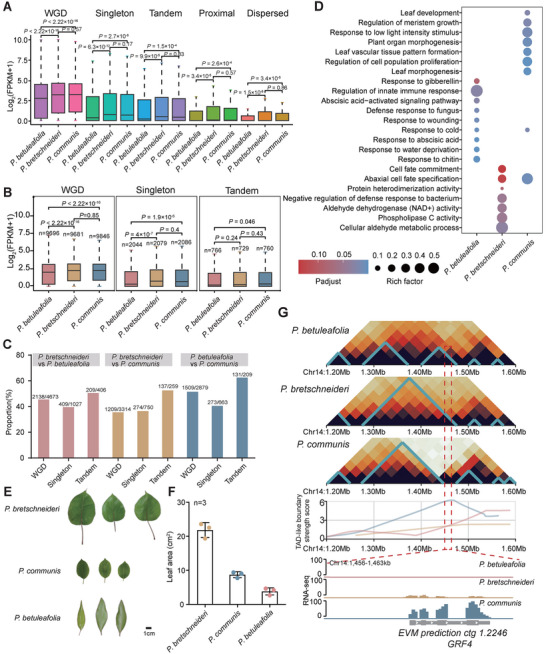
Distribution characteristics of genes associated with WGDs in the 3D genomes of *P. bretschneideri*, *P. betuleafolia*, and *P. communis*. (A) Expression levels of WGD (*n* = 23812), singleton (*n* = 5201), tandem duplicated (*n* = 1963), proximal (*n* = 1632), and dispersed (*n* = 8442) genes (Wilcoxon rank‐sum test). (B) Expression levels of WGD, singleton, and tandem genes in TAD‐like boundaries among *P. bretschneideri*, *P. betuleafolia*, and *P. communis*. The value (n) displayed below each *P‐*value indicated the number of genes in each group. (C) The proportion of WGD, Singleton and Tandem duplicated genes with expression differences in TAD‐like boundaries across different species. (D) The GO pathway analysis of WGD genes in TAD‐like boundaries among *P. bretschneideri*, *P. betuleafolia*, and *P. communis*. (E) Pictorial presentation of leaf and mature fruit phenotypes of three pear species (*n* = 3). Data are shown as mean ± s.e. (F) Histogram of leaf area of three pear species. (G) The example of differentially expressed WGD gene (*EVM prediction ctg1.2246, GRF4*) in TAD‐like boundaries among *P. bretschneideri*, *P. betuleafolia*, and *P. communis*, with Hi‐C maps (25 *K*b resolution) shown above, TAD‐like boundary score, RNA‐seq signals shown below. The *P‐*values in Figure 6 A and B were adjusted for multiple comparisons using the Benjamini‐Hochberg procedure (FDR) following Wilcoxon's rank‐sum tests.

Whole‐genome duplication (WGD) and resulting genetic redundancy are key drivers of plant evolution [46]. To investigate the potential correlation between WGD and the 3D chromatin structure of the pear genome, WGD associated genes, small scale duplicated genes (tandem, proximal, and dispersed duplicated genes), and single‐copy genes were identified using MCScanX [47]. Our analysis revealed that WGD genes exhibited greater expression levels than small scale duplicated genes and single copy genes across all three species (Figure 6A). The expression levels of all types of duplicated genes in the cultivated pears *P. bretschneideri* and *P. communis* were significantly higher compared to those in the wild pear *P. betuleafolia* (Figure 6A, *p* <0.001, Wilcoxon rank‐sum test). The mean FPKM values of proximal and distributed duplicated genes were around zero. Consequently, we focused on the 3D chromatin organizations of WGD, singleton, and tandem genes. When comparing the expression of WGD, singleton, and tandem genes in TAD‐like boundaries across the three species, we observed that WGD and singleton genes in the cultivated *P. bretschneideri* and *P. communis* pears exhibited significantly higher expression levels compared to the wild pear *P. betuleafolia* (Figure 6B, Wilcoxon rank‐sum test). This trend was consistent with the overall gene expression patterns throughout the genome. However, the tandem duplicated genes showed differential expression between *P. betuleafolia* and *P. communis* (Figure 6B, Wilcoxon rank‐sum test), but no differences were observed between these two and *P. bretschneideri* (Figure 6B, Wilcoxon rank‐sum test). When examining duplicated gene expression within TAD‐like boundaries across the three species, WGD and singleton genes in *P. bretschneideri* and *P. communis* consistently showed significantly higher expression than those in the wild species *P. betuleafolia*, and these patterns were unaffected by changes in replicates or step size (Figure S17).

We also assessed whether genes from different duplication modes (WGD, tandem duplicate, singleton) exhibit distinct propensities for expression changes upon TAD reorganization. Between *P. bretschneideri* and *P. betuleafolia*: WGD genes, 45.75% (2,138/4,673); singleton genes, 39.82% (409/1,027); tandem duplicate genes, 51.48% (209/406) (Figure 6C). Between *P. bretschneideri* and *P. communis*: WGD genes, 36.48% (1,209/3,314); singleton genes, 36.53% (274/750); tandem duplicate genes, 52.90% (137/259). Between *P. betuleafolia* and *P. communis*: WGD genes, 52.41% (1,509/2,879); singleton genes, 41.18% (273/663); tandem duplicate genes, 62.68% (131/209) (Figure 6C). These results indicate that tandem duplicate genes are the most responsive to TAD boundary alterations, while WGD and singleton genes also exhibit substantial sensitivity.

Using Gene Ontology (GO) analysis, we annotated and classified WGD, singleton, and tandem genes located in TAD‐like boundaries. The results revealed that WGD genes were significantly enriched in biological processes such as ‘Leaf development’, ‘Abaxial cell fate specification’ and hormone signaling pathways (including auxin, gibberellin, and jasmonic acid), which are closely associated with leaf development and stress response (Figure 6D). Upon examining the phenotypes of three pear leaves, we saw that the leaves of ‘Duli’ exhibit a small and slender shape, whereas those of ‘Dangshansuli’ had a larger, rounded form. The ‘Early red Doyenne du Comice’ possesses small, round leaves (Figure 6E). ‘Dangshansuli’ has the largest leaf area, followed by ‘Early Red Doyenne du Comice,’ and the smallest is the wild species ‘Duli’ (Figure 6F). These differentially expressed WGD genes in TAD boundaries may be related to leaf shape and size. For example, the WGD gene *GRF4* (*EVM prediction ctg 1.2246*), which is located on chromosome 14 (Chr14: 1,459,225–1,462,183), resides within the TAD‐like domain interior regions in *P. bretschneideri* and *P. betulaefolia*, and is positioned at the TAD‐like boundary in *P. communis* (Figure 6G). Additionally, its expression level in *P. communis* is higher than in *P. bretschneideri* and *P. betulaefolia*. GRF4 has been reported to promote cell division during early leaf development by activating cell proliferation‐related genes (e.g., PtoHB21), while coordinating developmental transitions by suppressing transition‐related factors (PtoLD). In poplar, PtoGRF9 (a homolog of GRF4) has been demonstrated to significantly affect leaf size by regulating cell proliferation [48]. In multiple plant species such as *Arabidopsis*, rice, and *Phalaenopsis*, homologs of GRF4 (AtGRF1/2, OsGRF1/2, PeGRF6) regulate leaf size and exhibit spatiotemporally specific expression patterns (e.g., high expression in apical meristems) [49, 50].

### 2.6 Population Analyses Reveal TAD‐Like Dynamics During Pear Domestication and Divergence

**FIGURE 7:**
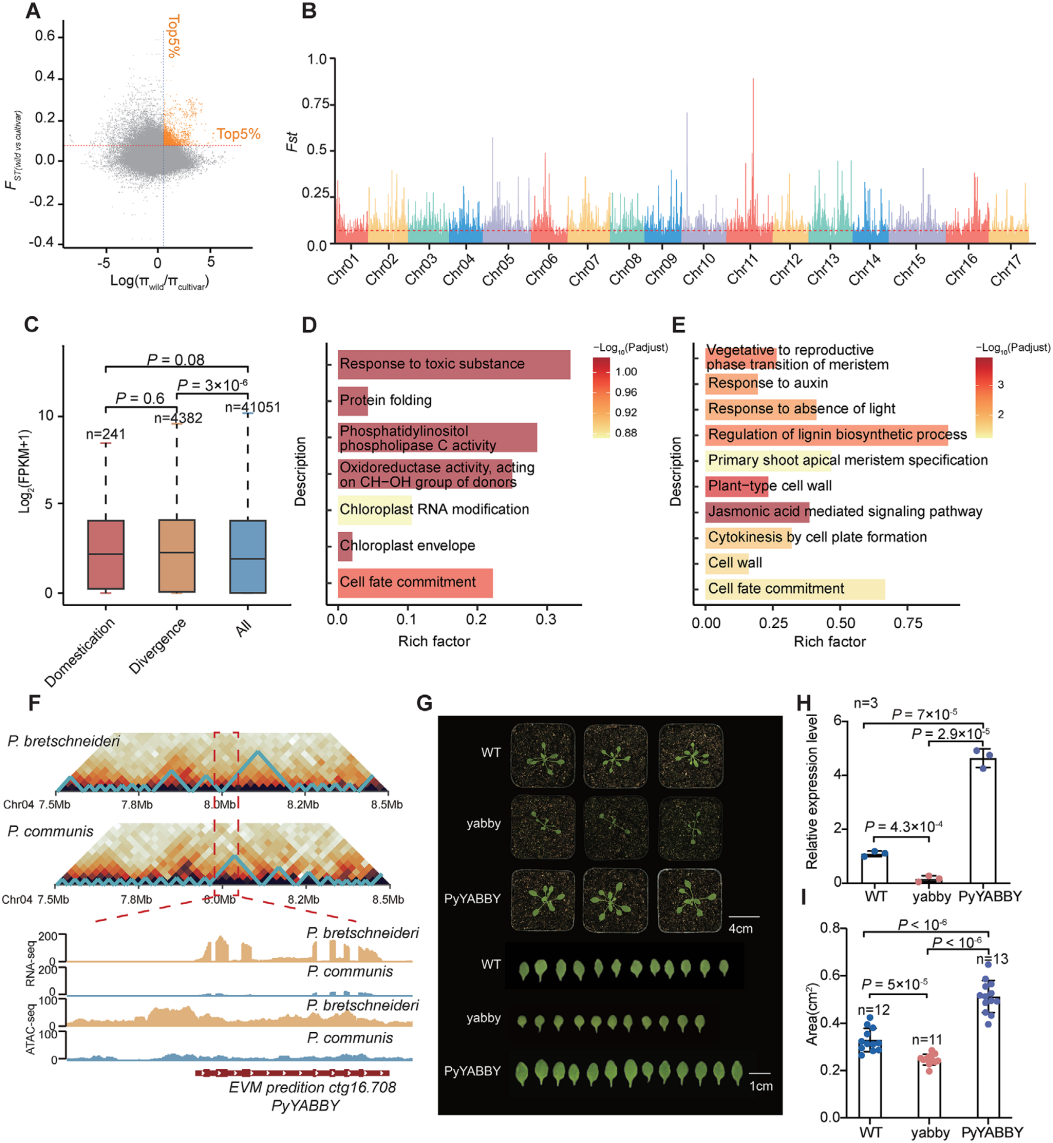
Identification of selective sweeps during pear domestication and differentiation. (A) The identification of selective sweeps during pear domestication. Each point represents a 10 kb slide window. Orange points represent candidate selective sweeps. The x‐axis depicts the π_wild_/_cultivar_ ratio; the y‐axis depicts the *F_ST_* value. (B) Identification of selective sweeps during pear differentiation. Orange points represent candidate selective sweeps. (C) Expression levels of genes associated with domestication (*n* = 241), differentiation (*n* = 4,382), and total genes (*n* = 41,051) in the ‘Dangshansuli’ pear. (D,E) GO annotation of genes in domestication and divergence regions around TAD‐like boundaries. (F) The heatmaps (25 kb resolution) of the *PyYABBY* gene, with RNA and ATAC‐seq signals below. (G) Growth phenotypes of the *PyYABBY* gene in pear transformed into *A. thaliana*. (H) Leaf area of wild‐type *A. thaliana*, yabby mutant *A. thaliana*, and PyYABBY‐complemented *A. thaliana* leaf size (*n* = 3). Data are shown as mean ± s.e. (I) YABBY expression patterns in wildtype (*n* = 12), *yabby* mutant (*n* = 11), and PyYABBY complementation infected (*n* = 13) *Arabidopsis thaliana* lines. Data are shown as mean ± s.e. Pairwise comparisons between groups were conducted using unpaired two‐tailed Student's t tests with a Bonferroni correction for multiple comparisons. The number (*n*) in each column represents biological replicates.

Excluding studies on fundamental genomic differences, our understanding of 3D chromatin structural variations that occurred throughout the domestication and divergence of pears is limited. To examine such variations, we used 22 cultivated Asian, 19 wild Asian, and 24 cultivated European pear accessions (Table S6) to identify genomic regions associated with domestication between the wild and cultivated Asian pears, as well as regions associated with divergence events between the Asian and European pears [31]. For wild and cultivated Asian pears, a total of 1155 slide windows were identified as candidate selection regions (*π_wild/cultivar_* > 3.15778 and *F_ST_* > 0.0799346), in which a total of 3.07 Mb of genome sequence and 1,305 genes were represented in these selection sweep regions (Figure 7A). In comparisons of the cultivated Asian and European pear accessions, more divergent slide windows were identified, with 27.67 Mb of genome sequence (*F_ST_* > 0.0761469; 23,822 windows) and 4,382 genes being represented in these windows (Figure 7B). Additionally, gene expression levels in regions associated with divergence in the Asian pear ‘Dangshansuli’ were significantly higher than all genome genes (Figure 7C, Wilcoxon rank‐sum test).

To determine whether variations in 3D organization were associated with gene expression during domestication and divergence events. The majority of them were conserved in the A–A (74% of domestication, 60% of divergence) and B–B (21% of domestication, 23% of divergence) compartments (Figure S18A,B). We further compared the selected regions and a number of genes that occurred in the domestication regions and TAD‐like boundaries between *P. bretschneideri* and *P. betuleafolia*. Similarly, we compared divergence regions and genes in the TAD boundaries between *P. bretschneideri* and *P. communis*. 115 domestication regions and 1819 divergent areas of TAD‐like boundaries. At TAD‐like boundaries, 234 genes were identified in the domestication regions, and 3,605 genes were identified in the region of divergence (Figure S18D). GO annotation showed that genes in domestication regions of TAD‐like boundaries were enriched in the ‘Response to toxic substance’ and ‘Cell fate commitment’ categories (Figure 7D). GO annotation showed that genes in divergence regions of TAD‐like boundaries were enriched in the ‘Regulation of lignin biosynthetic process', ‘Cell fate commitment’, and ‘Jasmonic acid‐mediated signaling pathway’ categories (Figure 7E).

In genome‐wide analysis, 47 genes were co‐located at the intersection of domestication regions and ‘Dangshansuli’‐specific TAD‐like boundaries, and 377 genes overlapped between divergent regions and ‘Dangshansuli’‐specific TAD‐like boundaries. The TF *PyYABBY* (*EVM prediction ctg 16.708*) (Figure 7F) was among these 377 genes. It is located in a genomic region overlapping a TAD‐like boundary that differs between ‘Dangshansuli’ and ‘Early red Doyenne du Comice'; as the WGD gene associated with ‘Cell fate commitment’, it has been verified to promote leaf development at an early stage. RT‐qPCR analysis revealed that the YABBY gene exhibited high expression in the leaf of ‘Dangshansuli’ pear and low expression in ‘Early red Doyene du Comice’ (Figure S19). We used Agrobacterium transformation to backfill the *PyYABBY* mutant and then evaluated the growth phenotype of the backfilled *A. thaliana*. The findings revealed that the expression levels of the *YABBY* gene in *yabby* mutants were much lower than those in wild‐type *A. thaliana* (Figure 7H). We found that the backfilled leaves were substantially larger than those of the *yabby* mutant at 10 days in *A. thaliana* (Figure 7G,I). Although these data do not establish causality between TAD‐like boundary variation and transcriptional divergence, they support a close association between domestication/divergence signals and local 3D chromatin organization at the TAD‐like level. By integrating population genomic signals with Hi‐C‐based 3D genome features and transcriptomes, domestication/divergence‐associated candidate genes were identified, which related to pear leaf traits and showed that these loci were associated with specific 3D chromatin features.

## 3 Discussion

The previous study elucidated the evolutionary history of pears, including the independent domestication of Asian and European pear species [31]. The study employed RNA sequencing analysis to investigate various wild, landrace, and improved pear (*Pyrus pyrifolia*) varieties to identify genetic modifications associated with domestication and improvement [51]. In this study, Hi‐C, PacBio HiFi long reads, ATAC‐seq, and RNA‐seq analysis were employed to construct Hi‐C maps of pear and produce a comprehensive analysis of the 3D chromatin structure of wild and cultivated Asian pear, as well as cultivated European pear species. We investigated TAD‐like domains and their boundaries, focusing on genes located within these genomic regions that were associated with leaf phenotypic traits. We also examined the structural features and genes implicated in leaf trait variation during domestication and differentiation.

Genomic structural variations (SVs) are major drivers of gene function evolution, genome architecture remodeling, and the emergence of domestication‐related traits [52, 53, 54]. Presence and absence variation (PAV) is the primary factor contributing to 3D genomic variations in soybeans. Roughly 55% of 3D genomic structural variations are associated with clear genetic differences, although half of these variations impact the expression of neighboring genes [40]. In this study, our analyses suggested that while most SVs were located in the interior of TAD‐like domains, those present at boundaries showed a positive correlation with boundary variation and the expression levels of neighboring genes. Future functional experiments are needed to validate this putative causal relationship. In the model plant *A. thaliana*, SVs are not simply randomly distributed in the 3D space of the genome, but tend to be distributed in regions with high degrees of chromatin interaction [55]. In cotton, SVs were preferentially located in TAD interior regions instead of TAD boundaries [41]. The same trend was observed in perennial pear plants. In addition, the presence of SVs and TAD‐like disruptions throughout the entire genome had a significant influence on the expression of orthologous genes in cotton. Our analysis revealed the presence of several TFs within SVs occurring near TAD‐like boundaries that were connected with leaf development and resistance, and these genes will undergo further verification.

In pepper, a chromatin accessibility map of 12 *Capsicum* accessions revealed that genome size variation is largely attributable to differential TE insertions. That study demonstrated that ACR evolution is closely associated with TEs, and interspecific comparisons further showed that SVs—both macro‐scale variants that reposition ACRs and micro‐scale variants that alter ACR sequence composition—can strongly influence ACR function, providing new insights into how SVs contribute to plant gene regulation [56]. Similarly, in tomato, SVs have been proposed as a key factor underlying the formation of cultivar‐specific accessible *cis*‐ACRs [57]. Accordingly, in our study, we quantified *cis*‐ACRs overlapping SVs and linked these SV‐associated regulatory regions to corresponding DEGs between species, generating a set of candidate SVs, enhancers, promoters, and key genes for prioritized functional validation (Figure S20).

In addition, TE also influenced on TAD organization. In cotton, the recent amplification of expressed TEs in *Gossypium rotundifolium* and *G. arboreum* may have contributed to the formation of lineage‐specific TAD boundaries following species divergence [58]. In soybean, an enrichment of non‐LTR retrotransposons was reported at TAD boundaries. By leveraging pan‐3D genome data, the study provided multiple lines of evidence that Gypsy LTRs and satellite repeats constitute private TAD boundaries [40].

Repetitive sequences constitute 53.1% of the assembled pear genome, consistent with the prevalence of whole‐genome duplication (WGD) in plants—a major evolutionary force driving speciation and genomic innovation. WGD events are typically accompanied by rapid structural reorganization and dynamic shifts in gene expression patterns [17, 26]. In this study, we compared the expression levels of WGD and small‐scale duplicated genes at TAD‐like boundaries; there was a significant discrepancy in the expression levels of WGD and single‐copy genes in TAD‐like boundaries between *P. bretschneideri* and *P. communis*. Between the wild pear *P. betuleafolia* and cultivated pears *P. bretschneideri* and *P. communis*, WGD genes in TAD‐like boundaries also exhibited significant differences. We speculated that WGD events may play a role in TAD‐like boundary formation, and that the observed divergence in TAD‐like domains among the three species was related to differences in their WGD gene expression levels.

Cultivated pears are derived from wild species, and there is a significant genetic difference between Asian and European pears. They have undergone substantial agronomic trait selection, including greater photosynthetic capacity, higher accumulation of starches and sugars, and enhanced resistance to biotic and abiotic stresses [31]. In soybeans, stronger artificial selection was observed for chromatin‐looped genes than genes without loops [17]. In cotton, a 120‐kb enhancer located upstream of the tubulin α‐3 (*TUA3*) gene had undergone domestication selection, probably associated with the differential expression of *TUA3* between cultivated and wild cotton accessions [59]. In our investigation, to explore the potential role of 3D genome architecture in pear evolution, we characterized the genomic distribution of domestication‐ and differentiation‐associated genes relative to TAD‐like boundaries. The expression levels of genes in genomic regions linked to divergence were significantly higher than in the areas associated with domestication events at TAD‐like boundaries. These genes are additionally linked to networks, including hormone signaling and cellular differentiation, which were closely associated with leaf development and resistance qualities.

Notably, PyYABBY, a WGD gene identified in TAD‐like boundary differentiation regions, was experimentally confirmed to enhance leaf size during early growth stages. YABBY transcription factors play important roles in multiple biological processes, including polarity establishment in plant leaves, the formation and development of reproductive organs, responses to plant hormone signals, resistance to stress, crop breeding, and agricultural production [60]. The role of YABBY genes in regulating leaf size is well‐established and evolutionarily conserved across plant species, while the underlying molecular mechanisms exhibit differences. YABBY genes modulate leaf size primarily by coordinating cell division and expansion processes. For example, in *Arabidopsis thaliana*, YABBY members integrate into networks that control cell proliferation and expansion, ultimately determining the final leaf size [61]. We selected *Arabidopsis thaliana* as the heterologous system because the availability of *YABBY* mutant lines (e.g., *yabby* mutants) enables direct testing of complementation and overexpression effects. Introducing pear *YABBY* into the mutant background produced clear and reproducible leaf phenotypes in 3–4 independent experiments across numerous transgenic lines, consistently demonstrating that *PyYABBY* promotes leaf enlargement. Moreover, for perennial fruit trees with low transformation efficiency, *Arabidopsis* provides an optimal platform for functional validation of leaf functional genes, balancing efficiency, feasibility, and experimental rigor [62, 63, 64].

In animals, high‐resolution single cell Hi‐C has revealed cell‐to‐cell heterogeneity in chromatin architecture and dramatic restructuring during cell differentiation [65]. In plants, while bulk methods like INT‐Hi‐C have identified tissue‐specific conformations, they mask cellular variation [66]. Techniques such as INT‐Hi‐C, which combined INTACT‐based cell‐type enrichment with Hi‐C, have successfully identified distinct chromatin conformations in endosperm and leaf tissues [67]. Recent single‐cell studies in rice and *Arabidopsis* have now resolved dynamic chromatin structures in gametes, zygotes, and male gametophytes, linking 3D reorganization to genome activation and cell‐type‐specific transcription, demonstrating the unique power of single‐cell approaches [68, 69]. In the future, applying single‐cell Hi‐C analysis to fruit tree tissues will enhance the resolution and accuracy of chromatin structure characterization.

## 4 Experimental Section

### 4.1 Pear Materials Preparation

Asian cultivated ‘Dangshansuli’ (*P. bretschneideri*) and Asian wild ‘Duli’ (*P. betuleafolia*) pear trees were maintained at the Shandong Agricultural University. European cultivated pear ‘Early red Doyene du Comice’ (*P. communis*) trees were maintained at the Yantai Academy of Agricultural Sciences, China. Fresh young leaves were sampled individually from each pear accession and immediately flash‐frozen in liquid nitrogen before nucleic acid extraction and preparation for Hi‐C, Pacbio HiFi long reads, ATAC‐seq, and RNA‐seq analyses.

Genomic DNA was extracted using the CTAB method for WGS. Following the manufacturer's recommendations, paired‐end DNA libraries with small inserts (∼500 bp) were constructed and sequenced using the USA‐based, Illumina HiSeq 2000 or HiSeq 4000 platforms. Reads having > 5% N (missing) bases and > 50% of bases with a quality score less than 5 were considered discarded to produce the clean read dataset.

### 4.2 The Methods of Genome Assembly of ‘Dangshansuli’ Pear

The PacBio HiFi reads and Hi‐C data were obtained from a previous study [32]. The genome of the ‘Dangshansuli’ pear was assembled using two approaches: hifiasm (v0.19.0) [70], which utilized PacBio HiFi and Hi‐C data. The resulting contigs were then anchored to chromosomes using ALLHIC (v0.9.8) [71] with Hi‐C data, followed by manual correction. To assess the assembly completeness and quality, NGS reads were aligned to the ‘Dangshansuli’ assembly using BWA [72]. SAMtools (v1.14) [73] was subsequently employed to calculate genome coverage and mapping rate. Additionally, genome completeness was assessed using BUSCO (v5.2.2) [74] with the eukaryotic_odb10 database. LAI (LTR Assembly Index) was calculated using LTR_retreiver [34]. Gene prediction and annotation followed the methods described in the same study [32].

### 4.3 Gene Function Annotation

Gene functions were predicted by aligning the protein sequences to the Swiss‐Prot [75] and NR [76] databases using BLAST search (with threshold E‐value ≤1e–5). Pfam [77] was used to identify conserved functional motifs. EggNOG [78] database was utilized to perform functional annotation of proteins, orthology assignments, and Gene Ontology (GO) term identification for all genes in ‘Dangshansuli’ genome. KEGG pathway annotations were obtained by searching against the KEGG database using KofamScan [79].

### 4.4 Hi‐C Library Preparation and Sequencing

Hi‐C libraries were constructed according to previous studies [80]. Briefly, ∼3 g pear leaf tissue was ground into powder in liquid nitrogen, and then incubated with nuclei‐isolation buffer (20 mM Hepes pH8.0, 250 mM Sucroses, 1 mM MgCl_2_, 5 mM KCl, 40% glycerol, 0.25% Triton X‐100, 1x β‐mercaptoethanol, 1x PMSF, 1x Roche cOmplete EDTA‐free) at 4°C for 15 min. After filtering through a 40 µm Cell Strainer, nuclei were collected by centrifugation and resuspended in cold 1x PBS buffer. Then, nuclei were cross‐linked for 15 min with 3% formaldehyde at 4°C and quenched with 0.375 M final concentration glycine for 5 min. Subsequently, the cross‐linked nuclei were permeated with 0.3% SDS at 65°C for 10 min. SDS were quenched by 1.8% Triton X‐100 at 37°C for 15 min. Chromatin was then digested with 100 U of MboI (NEB) restriction enzyme for 2 h at 37°C. DNA ends were labeled with biotin‐14‐dCTP (Invitrogen) by incubating with DNA Polymerase I, Large Klenow Fragment (NEB) at 37°C for 45 min, after which the reaction was inactivated by incubation at 65°C for 20 min. DNA ligation was performed by 50U T4 DNA ligase (Thermo) at 16°C for 4 h. Afterward, proteinase K was added to the sample and incubated at 65°C overnight to reverse the cross‐linking. DNA was extracted using the Qiagen DNeasy Plant Mini Kit (Qiagen, #69106), following the manufacturer's instructions. The DNA was then purified, and unligated biotin‐labeled dCTP ends were removed using T4 DNA Polymerase (NEB) with dATP and dTTP. Purified DNA was sheared to 300‐ to 500‐bp fragments and was further end‐repaired, A‐tailed, and adaptor‐added using NEBNext Ultra II DNA Library Prep Kit for Illumina (NEB, #E7805L). Biotin‐labeled DNA fragments were separated using Streptavidin C1 beads (Life Technologies) followed by PCR amplification. Finally, the Hi‐C libraries were sequenced on the Illumina Nova‐seq6000 platform (San Diego, CA, USA). The Hi‐C experiment of each sample was performed with two biological replicates. The sequencing depths estimated by clean data were 206× (*P. bretschneideri* biological replicate 1), 216× (*P. bretschneideri* biological replicate 2), 209× (*P. betuleafolia* biological replicate 1), and 216× (*P. betuleafolia* biological replicate 2), 206× (*P. communis* biological replicate 1), 216× (*P. communis* biological replicate 2).

### 4.5 Hi‐C Reads Mapping and Normalization

Trimmomatic [81] software was used to filter low‐quality reads and remove adapter sequences from the raw Hi‐C data (trimmomatic PE ‐threads 40 ‐phred33 ‐trimlog logfile DS‐leaf_1.fq DS‐leaf_2.fq DS_R1.clean.fastq DS_unpair_1.fq DS_R2.clean.fastq DS_unpair_2.fq ILLUMINACLIP: TruSeq3‐PE.fa:2:30:10 LEADING:3 TRAILING:3 SLIDINGWINDOW:4:15 MINLEN:36). Clean reads were mapped to the ‘Dangshansuli’ genome using Juicer (version 1.19) [82] (bash /bin/juicer‐1.6/CPU/juicer.sh ‐d DS ‐s MboI ‐p dangshan_hifi_chrom.size ‐y dangshan_hifi_chr_MboI.txt ‐z dangshan_hifi_chr.fa). The iterative correction and eigenvector decomposition (ICE) method was used to normalize the Hi‐C contact matrices at varying resolutions (5, 25, 50, and 100 kb) for downstream analyses. The Hi‐C read contact matrix was visualized using the Juicebox (version 1.11), HiCexplorer [83], and FAN‐C [84] software packages. Hic2cool (https://github.com/4dn‐dcic/hic2cool) and cooler (https://github.com/open2c/cooler) were used to convert. hic file into. cool txt. HiCCUPS and APA analysis algorithms within the Juicer toolkit to investigate chromatin loops across the entire genome. The detailed parameters are as follows: 1) java ‐jar juicer_tools.jar hiccups –cpu ‐k KR ‐r 10,000 DS.hic ./DS_10kb_hiccups; 2) java ‐jar juicer_tools.jar ‐r 10,000 ‐k KR DS.hic merged_loops.bedpe ./DS_APA. We used the hicCorrelate program in HiCExplorer [83] to assess the correlation between biological replicates of the two Hi‐C datasets.

The duplicate valid pair files were generated by Juicer and then merged with the Juicer script ‘mega.sh’ (mega.sh ‐g dangshan_hifi.chrom.sizes ‐s MboI). The resulting merged contact matrix was employed for compartment (A/B) identification and TAD‐like structure calling. Cworld‐dekker package [85] (https://github.com/dekkerlab/cworld‐dekker) was used for Compartments A/B identification at 50 kb resolution (perl matrix2compartment.pl ‐i DS_50kb_matrix.txt; python matrix2EigenVectors.py ‐i DS_50kb_matrix.zScore.matrix.gz ‐r DS_gene.bed). Chromatin loops (*p* value < 0.005, contacts ≥ 10) were identified using FitHiC2 [86] at a 5 kb resolution. The valid interaction data used for loop identification were generated by merging raw data from two biological replicates and processing them through the HiC‐Pro [87] pipeline.

### 4.6 Analysis of Topologically Associated Domain‐Like Structure (TAD‐Like Structure)

TAD‐like identification in 25 kb resolution was also using Cworld packages (perl matrix2insulation.pl ‐i DS_25kb_matrix.txt ‐o DS_25kb_insulation –is 100 000 –ids 50 000; perl insulation2tads.pl ‐i DS_hic_25 kb.insulation ‐b DS_hic_25 kb.boundaries ‐o DS_hic_25kb_tad). The boundary strength score in a bed file was also calculated using this program. Conserved TAD‐like structure boundaries between two species were defined as those with a maximum boundary change of two times the resolution (50 kb). Conserved boundaries were defined as those with two conserved boundaries. This method references the comparative analysis of TAD‐like structures in cotton [88]. Conserved TAD‐like structure boundaries between two species were considered conserved if the boundary change did not exceed two times the resolution (50 kb).

### 4.7 Transposable Element Prediction

TEs were identified and annotated using the EDTA tool [33] with the parameters ‘–sensitive 1 –anno 1 –evaluate 1 –force 1’.

### 4.8 Identification of WGD Events in the ‘Dangshansuli’ Genome

For whole‐genome duplication (WGD) gene information from MCScanX [47], specific parameters are as follows: 1) makeblastdb ‐in dangshansuli_pep.fa ‐dbtype prot ‐parse_seqids ‐out dangshansuli_hifi; 2) blastp ‐query dangshansuli_pep.fa ‐db index/ dangshansuli ‐out dangshansuli.blast ‐evalue 1e‐5 ‐outfmt 6 ‐num_alignments 5; 3) MCScanX dangshansuli; 4) duplicate_gene_classifier dangshansuli. The script was written in Python for converting intermediate file formats.

### 4.9 ATAC‐Seq Library Construction and Sequencing

Pear leaf tissues were ground into powder in liquid nitrogen, and then incubated with lysis buffer (10 mM Tris‐HCl pH 8.0, 10 mM NaCl, 3 mM MgCl_2_, 0.25 M sucrose, 1% Triton X‐100, 10 mM DTT, 1 × Protease Inhibitor Cocktail) at 4°C for 15 min [89]. After filtration with a 40 µm cell strainer, the cell solution was slowly transferred to the top of 60% personal solution and 2.5 M sucrose, and centrifuged at 1,800 × *g* for 20 min. The intermediate phase of the centrifuged liquid was extracted and then diluted with 10 mL RSB buffer (10 mM Tris‐HCl pH 8.0, 10 mM NaCl, 3 mM MgCl_2_). Cell nuclei pellets were harvested by centrifugation at 1000 × *g* for 10 min and washed with RSB buffer once. Approximately 50,000 nuclei were added to the transposition reaction solution to perform tagmentation (Vazyme #TD501). Tn5 transposed DNA was purified by AMPure DNA magnetic beads and PCR amplification (72°C for 3 min, 98°C for 30 s, and thermocycling at 98°C for 15 s, 60°C for 30 s, and 72°C for 3 min, followed by 72°C for 5 min). The final qualified library was sequenced on the Illumina Novo‐seq6000 platform (San Diego, CA, United States).

ATAC‐seq library construction of each sample was performed with two biological replicates. The sequencing depths estimated by raw data were 50× (*P. bretschneideri* biological replicate 1), 52× (*P. bretschneideri* biological replicate 2), 52× (*P. betuleafolia* biological replicate 1), 48× (*P. betuleafolia* biological replicate 2), 49× (*P. communis* biological replicate 1) and 55× (*P. communis* biological replicate 2).

Bowtie2 v2.2.8 [90] software was used to map the clean reads from each sample to the reference genome using the parameter ‘‐X 2000’ and reads aligning to the mitochondria or chloroplasts were filtered. Duplicates were removed using Picard (https://github.com/broadinstitute/picard). Bam files were converted to bed formats using BEDTools [91]. BigWig files were generated by BamCoverage with 1x average coverage in deepTools [92] and visualized using IGV [93]. MultiBigwigSummary and plotCorrelation in deepTools [92] were used to calculate and summarize the correlation coefficient between two replicates of ATAC‐seq data and to generate a plot.

For ACR identification, reads from the ATAC‐seq and input library were down‐sampled to the same level using Picard. Peaks were called by MACS2 (version 2.1.2) [94] with the parameters ‘–shift ‐75 –extsize 150 –nomodel ‐B –SPMR ‐g 500,000,000 –keep‐dup all’. ACRs were described as peaks positioned 2 kb upstream of transcription start sites within the gene body, and 500 bp downstream of transcription termination sites.

### 4.10 Peak‐Related Gene Annotation and Analysis

According to genomic location information and gene annotations, peak‐related genes were confirmed using ChIPseeker v1.16.1 [95]. The distribution of peaks in different genome regions (including promoters, 5’UTRs, 3’UTRs, exons, introns, downstream regions, and intergenic regions) was also performed. Pathway enrichment analysis identified significantly enriched metabolic pathways or signal transduction pathways in peak‐related genes compared with the whole genome background.

### 4.11 Identification of SVs using Pacbio HiFi Long Reads of *P. betuleafolia* and *P. Communis*

The sequencing depths estimated from clean PacBio HiFi long reads were 47× for *P. bretschneideri*, 45× for *P. betulifolia*, and 40× for *P. communis*. The PacBio HiFi long reads were mapped to the ‘Dangshansuli’ pear genome. The parameters used for Minimap2 [96] were as follows: ‘minimap2 ‐ax map‐hifi pear.fasta ‐t 50 dangshan_pac.fq > dangshan_pac.sam’. Aligned files in SAM format were converted to BAM format, then sorted using SAMtools (v1.9) [73]. After mapping reads to the reference genome, SVs were identified from the processed BAM files. CuteSV [97] was chosen for SV detection. The parameters used for CuteSV were: ‘cuteSV duli.sorted.bam dangshansuli.fasta cutesv.vcf –max_ cluster_bias_INS 100 –diff_ratio_merging_INS 0.3 –max_cluster_bias_DEL 200 –diff_ratio_merging_DEL 0.5 ‐t 30’.

We classified the SVs according to their positional relationship concerning TAD‐like boundaries. In brief, we classified SVs into three categories: ABA SVs were defined as SVs spanning the whole length of one boundary, PBA SVs were defined as SVs spanning part of the length of one boundary, and NBA SVs were defined as SVs within TADs [40].

### 4.12 RNA Extraction and Sequencing

Total RNA was extracted using the Plant Total RNA Isolation Kit Plus (World's Foregene) according to the manufacturer's instructions. RNA quality was assessed by electrophoresis on a 1% agarose gel. RNA purity was checked using the NanoPhotometer spectro‐ photometer (IMPLEN, CA, USA). RNA concentration was measured using a Qubit RNA Assay Kit in a Qubit 2.0 Fluorometer (Life Technologies, CA, USA). RNA integrity was assessed using the RNA Nano 6000 Assay Kit for the Bioanalyzer 2100 system (Agilent Technologies, CA, USA).

For each sample, three biological replicates were performed. A total of 9 independent libraries named DS‐1, DS‐2, DS‐3, DL‐1, DL‐2, DL‐3, ZH‐1, ZH‐2 and ZH‐3 for young leaves were prepared following the instructions of the NEBNext Ultra RNA Library Prep Kit for Illumina (NEB, USA). A total of 3 µg RNA per sample was used as input material for the RNA sample preparation. The libraries were sequenced on an Illumina HiSeq 2000 platform at the Beijing Genomics Institute (BGI, Shenzhen, China) and raw reads were generated in 125‐bp paired‐end format.

The Trim_galore (version 0.32) program (https://www.bioinformatics.babraham.ac.uk/projects/trim_galore/) was used to filter low‐quality reads, with parameters ‘‐q 25 –phred33 –length 36 ‐e 0.1 –stringency 3 –paired’. The clean reads were mapped to the ‘Dangshansuli’ reference genome using the Hisat alignment tool [98]. Differential gene expression analysis between different species (biological replicates per group) was implemented using the DESeq R package [99]. Genes with *P* ≤ 0.05 found by DESeq were set as the threshold for significantly differential expression. The KOBAS [100] program was used to test the statistical enrichment of DEGs in the KEGG pathway.

### 4.13 Detection of Selective Sweeps during Domestication and Divergence

We used 65 pear accessions, including 19 wild Asian, 22 cultivated Asian, and 24 cultivated European accessions, to identify selective signals in pear [31] (Table S6). The average sequencing depth of these samples was 10×. The methods were according to genome‐wide association studies of pear [101].

The raw reads were filtered using Trimmomatic and BWA was used for mapping clean reads to ‘Dangshansuli’ genome. Picard was used to remove duplicated reads from bam files. Gatk3 (version 3.8‐1‐0) was used to call SNPs, including HaplotypeCaller, SelectVariants, VariantFiltration, CombineVariants.

SNPs with missing rates of less than 0.3 in all pear accessions were deemed common SNPs for selection sweep analysis using Variant Call Format (VCF) tools [102]. To identify regions with selective signals, the π ratio (π_wild/cultivar_) and *F_ST_* values were calculated in 10 kb slide windows across the entire pear genome. 10 kb slide windows with significant selective signals were identified using the following criteria: top 5% of *F_ST_* and 5% of the π ratio. Genes with selective signals identified from these regions were selected as candidate genes.

### 4.14 *Arabidopsis* Transformation

*Agrobacterium tumefaciens* containing the vector 35S::*YABBY*‐GFP was transformed into *A. thaliana* Col‐0 plants using the floral dip method. Expression levels of *YABBY* in the primary inflorescence stem of two‐week‐old T_1_ plants were analyzed by semi‐quantitative RT‐PCR using primers listed in Table S7. Subsequently, the plants were transplanted in a glasshouse with a 16 h photoperiod. The height and diameter of the primary inflorescence stem were determined for each transgenic line and the WT control at 10 days post‐germination. [Correction added on 13 May 2026 after first online publication: supporting information table citation is updated in section 4.14.]

### 4.15 Gene Expression Analysis Using qPCR

cDNA was synthesized using a one‐step gDNA removal and cDNA synthesis kit (Transgen). qPCR was performed using the LightCycler 480 SYBR GREEN Master system (Roche). Primers were shown in Table S7. The analysis was conducted using three biological and three technical repeats. Relative expression levels of each gene were calculated using the 2^−ΔΔCp^method. *PbrGAPDH* and *AtACTIN*/*AtEF1α* were used as reference genes for pear and *A. thaliana*, respectively.

### 4.16 Statistical Analysis

Statistical analyses were performed using R (version 3.6) and Python (version 2.7). Specifically, enrichment analyses for Gene Ontology (GO) and KEGG pathways were conducted using the hypergeometric test, with false discovery rate (FDR) correction for multiple comparisons. An FDR‐adjusted *p*‐value (*q*‐value) of < 0.05 was considered statistically significant for enrichment. To quantify the effect sizes, we used two metrics:

$$\mathrm{Fold}-\mathrm{enri}\mathrm{chme}\mathrm{nt}:\;\left(\mathrm{FE}=\frac{\mathrm{obse}\mathrm{rved}}{\mathrm{mean}\left(\mathrm{perm}\mathrm{uted}\right)}\right)$$

and

$$Z-\mathrm{score}:\left(Z=\frac{\mathrm{obse}\mathrm{rved}-\mathrm{mean}\left(\mathrm{perm}\mathrm{uted}\right)}{\mathrm{sd}\left(\mathrm{perm}\mathrm{uted}\right)}\right).$$

For evaluation FE > 1 indicated enrichment and FE < 1 indicated depletion relative to random expectation. Z > 1.96 considered to reflect potential enrichment. For statistical thresholds, *P*‐value<0.05 was considered as significant.

For comparisons of continuous variables across the three species—including interaction frequencies, TAD‐like boundary strength scores, and gene expression levels—the non‐parametric Wilcoxon rank‐sum test was employed. Differences in leaf area and RT‐qPCR data were assessed using two‐tailed Student's *t*‐tests. All tests were implemented in R, and a *P*‐value < 0.05 was defined as the threshold for significance.

## Author Contributions

**J.W**. conceived and designed the project. **Y.Y.L**. analyzed the data and drafted the manuscript. **C.H.H**., **Y.S.X**., and **G.Y.Y**. conducted functional validation tests of relevant genes. **B.B.S**., **M.Y.S**., and **R.Z.W**. gave suggestions on the analysis. **C.X**., **S.Z.X**., and **J.M.L**. provided the samples. **J.W**. revised the manuscript. All authors have read and approved the final manuscript.

## Funding

This work was funded by the National Natural Science Foundation of China (U24A20415, 32502631), the Earmarked Fund for China Agriculture Research System (CARS‐28), Jiangsu Agricultural Science and Technology Innovation Fund (CX(23)3016), the Advanced Talents Research Foundation of Shandong Agricultural University, and the China Postdoctoral Science Foundation (2025M773736).

## Conflicts of Interest

The authors declare no conflicts of interest.

## Supporting information




**Supporting File 1**: advs75472‐sup‐0001‐FiguresS1‐S20.zip.

**Supporting File 2**: advs75472‐sup‐0002‐TablesS1‐S7.zip.
[Correction added on 13 May 2026 after first online publication: supporting information file 2 is updated.]

## Data Availability

The Hi‐C, ATAC‐seq, PacBio HiFi long reads, and RNA‐seq raw data generated in this study are available at the National Genomics Data Center, Chinese Academy of Sciences (https://ngdc.cncb.ac.cn/), under the accession number PRJCA063483. The files for genome assembly and annotation in this study are also available in the Pear Genomics Database (http://pyrusgdb.sdau.edu.cn/). All data are publicly available. Source data are provided with this paper.
