# Supplementary material for: 3D Chromatin Architecture Provides Insights Into Leaf Trait Variation Among Pear Species
Source: Adv Sci (Weinh). 2026 May 12;13(41):e19321. doi: 10.1002/advs.202519321 (PMC13335592; doi:10.1002/advs.202519321)
Supplement: Supplementary file 1 — Supporting File 1: advs75472‐sup‐0001‐FiguresS1‐S20.zip. [file ADVS-13-e19321-s002.zip › advs75472-sup-0008-FigureS8.pdf]

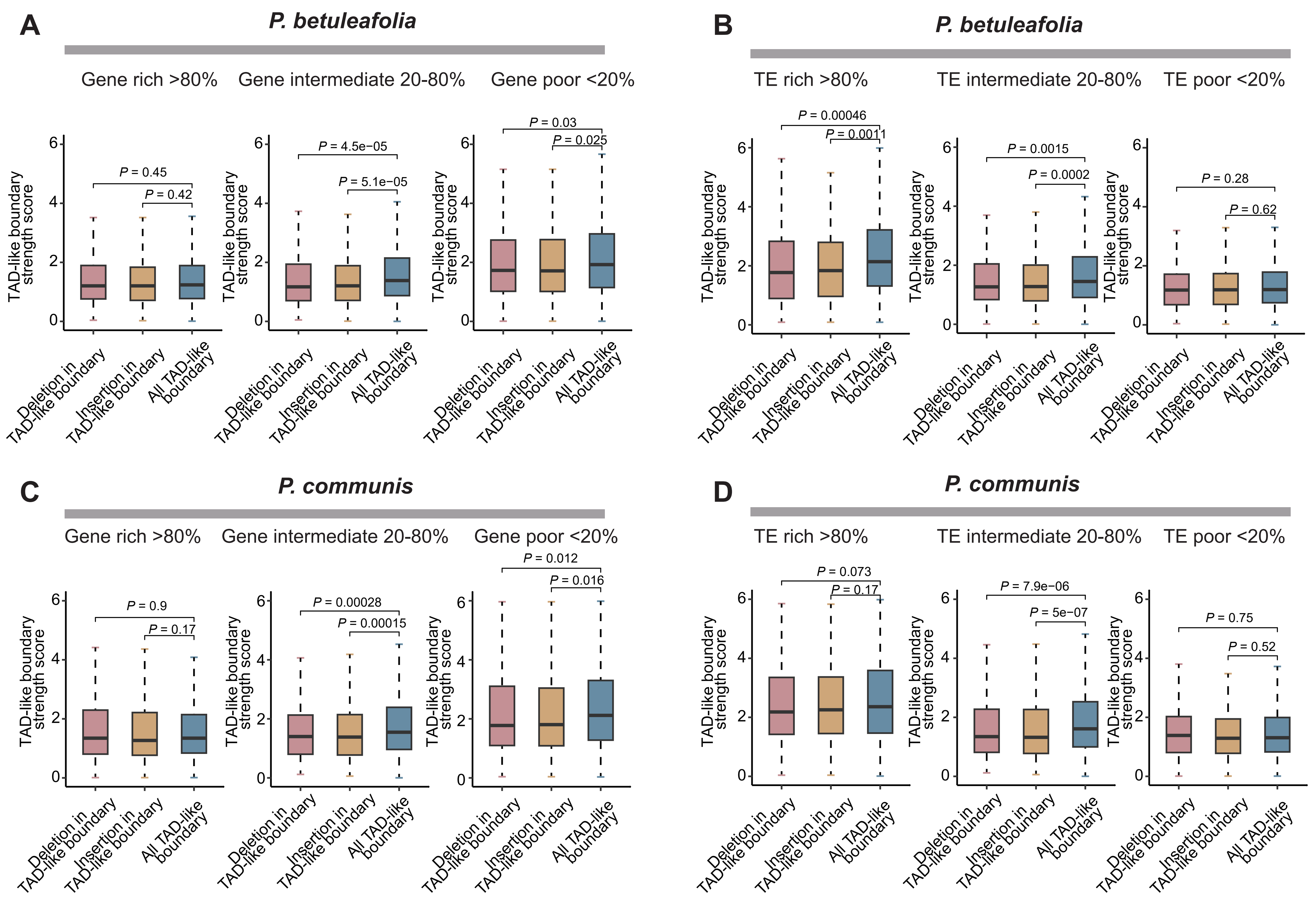
A
P. betuleafolia
Gene rich >80%
Gene intermediate 20-80%
Gene poor <20%
P = 0.45
P = 0.42
P = 4.5e−05
P = 5.1e−05
P = 0.03
P = 0.025
B
P. betuleafolia
TE rich >80%
TE intermediate 20-80%
TE poor <20%
P = 0.00046
P = 0.0011
P = 0.0015
P = 0.0002
P = 0.28
P = 0.62
C
P. communis
Gene rich >80%
Gene intermediate 20-80%
Gene poor <20%
P = 0.9
P = 0.17
P = 0.00028
P = 0.00015
P = 0.012
P = 0.016
D
P. communis
TE rich >80%
TE intermediate 20-80%
TE poor <20%
P = 0.073
P = 0.17
P = 7.9e−06
P = 5e−07
P = 0.75
P = 0.52
TAD-like boundary strength score
Deletion in TAD-like boundary
Insertion in TAD-like boundary
All TAD-like boundary
