# Supplementary material for: 3D Chromatin Architecture Provides Insights Into Leaf Trait Variation Among Pear Species
Source: Adv Sci (Weinh). 2026 May 12;13(41):e19321. doi: 10.1002/advs.202519321 (PMC13335592; doi:10.1002/advs.202519321)
Supplement: Supplementary file 1 — Supporting File 1: advs75472‐sup‐0001‐FiguresS1‐S20.zip. [file ADVS-13-e19321-s002.zip › advs75472-sup-0009-FigureS9.pdf]

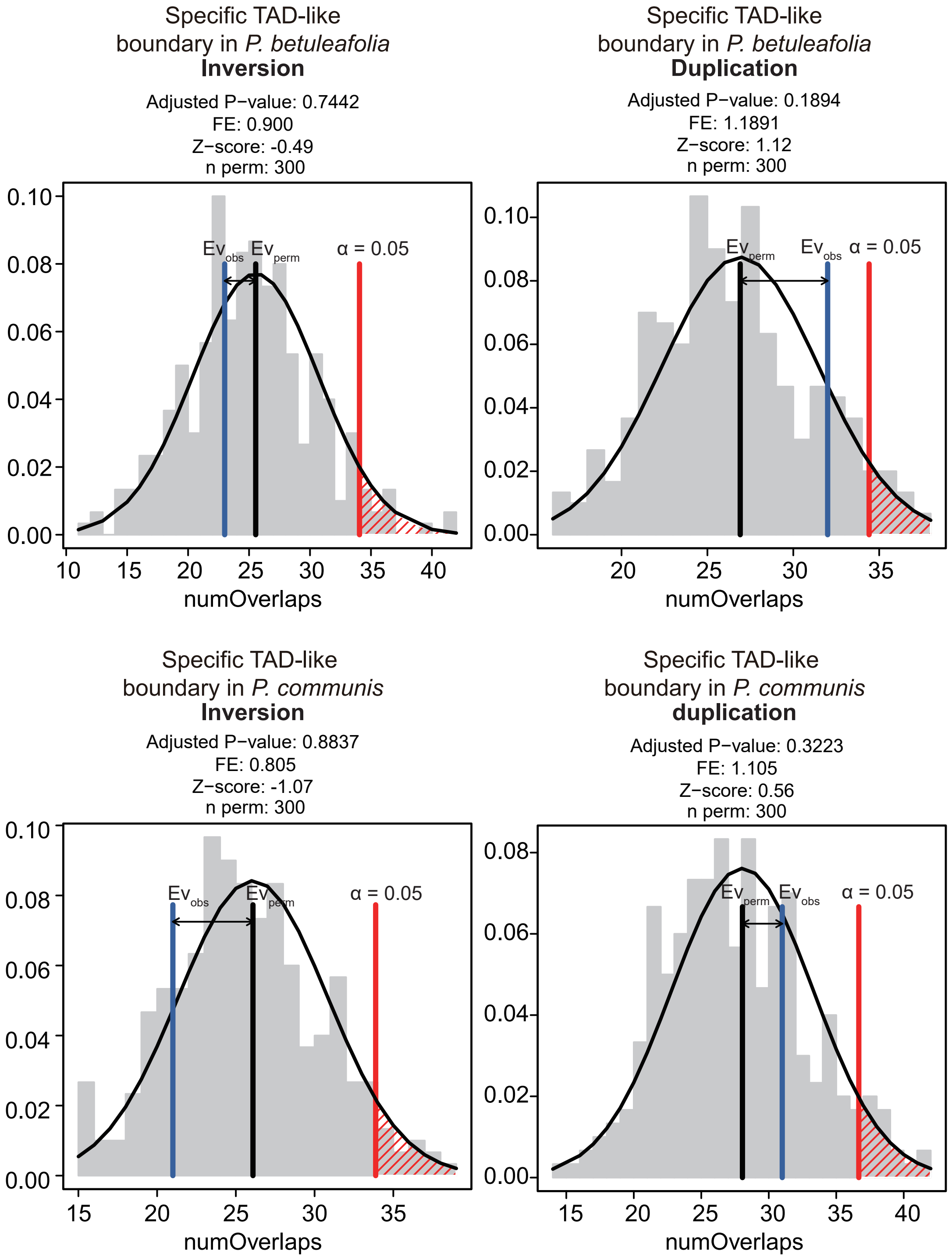
Specific TAD-like boundary in *P. betuleafolia*
Inversion
Adjusted P−value: 0.7442
FE: 0.900
Z−score: -0.49
n perm: 300
Ev obs
Ev perm
α = 0.05
0.10
0.08
0.06
0.04
0.02
0.00
10 15 20 25 30 35 40
numOverlaps
Specific TAD-like boundary in *P. betuleafolia*
Duplication
Adjusted P−value: 0.1894
FE: 1.1891
Z−score: 1.12
n perm: 300
Ev perm
Ev obs
α = 0.05
0.10
0.08
0.06
0.04
0.02
0.00
20 25 30 35
numOverlaps
Specific TAD-like boundary in *P. communis*
Inversion
Adjusted P−value: 0.8837
FE: 0.805
Z−score: -1.07
n perm: 300
Ev obs
Ev perm
α = 0.05
0.10
0.08
0.06
0.04
0.02
0.00
15 20 25 30 35
numOverlaps
Specific TAD-like boundary in *P. communis*
duplication
Adjusted P−value: 0.3223
FE: 1.105
Z−score: 0.56
n perm: 300
Ev perm
Ev obs
α = 0.05
0.08
0.06
0.04
0.02
0.00
15 20 25 30 35 40
numOverlaps
