# Supplementary material for: 3D Chromatin Architecture Provides Insights Into Leaf Trait Variation Among Pear Species
Source: Adv Sci (Weinh). 2026 May 12;13(41):e19321. doi: 10.1002/advs.202519321 (PMC13335592; doi:10.1002/advs.202519321)
Supplement: Supplementary file 1 — Supporting File 1: advs75472‐sup‐0001‐FiguresS1‐S20.zip. [file ADVS-13-e19321-s002.zip › advs75472-sup-0010-FigureS10.pdf]

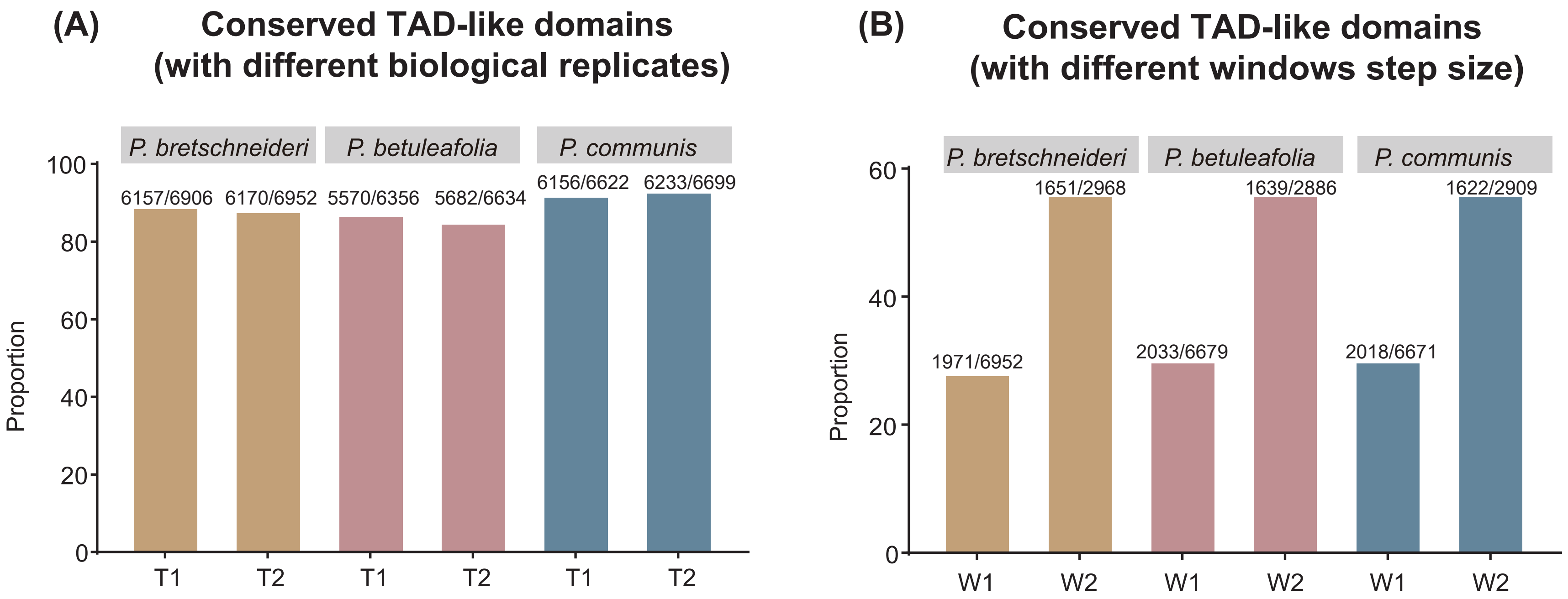

(A)
Conserved TAD-like domains
(with different biological replicates)
P. bretschneideri
P. betuleafolia
P. communis
Proportion
100
80
60
40
20
0
6157/6906
6170/6952
5570/6356
5682/6634
6156/6622
6233/6699
T1
T2
T1
T2
T1
T2
(B)
Conserved TAD-like domains
(with different windows step size)
P. bretschneideri
P. betuleafolia
P. communis
Proportion
60
40
20
0
1971/6952
1651/2968
2033/6679
1639/2886
2018/6671
1622/2909
W1
W2
W1
W2
W1
W2
