# Supplementary material for: 3D Chromatin Architecture Provides Insights Into Leaf Trait Variation Among Pear Species
Source: Adv Sci (Weinh). 2026 May 12;13(41):e19321. doi: 10.1002/advs.202519321 (PMC13335592; doi:10.1002/advs.202519321)
Supplement: Supplementary file 1 — Supporting File 1: advs75472‐sup‐0001‐FiguresS1‐S20.zip. [file ADVS-13-e19321-s002.zip › advs75472-sup-0011-FigureS11.pdf]

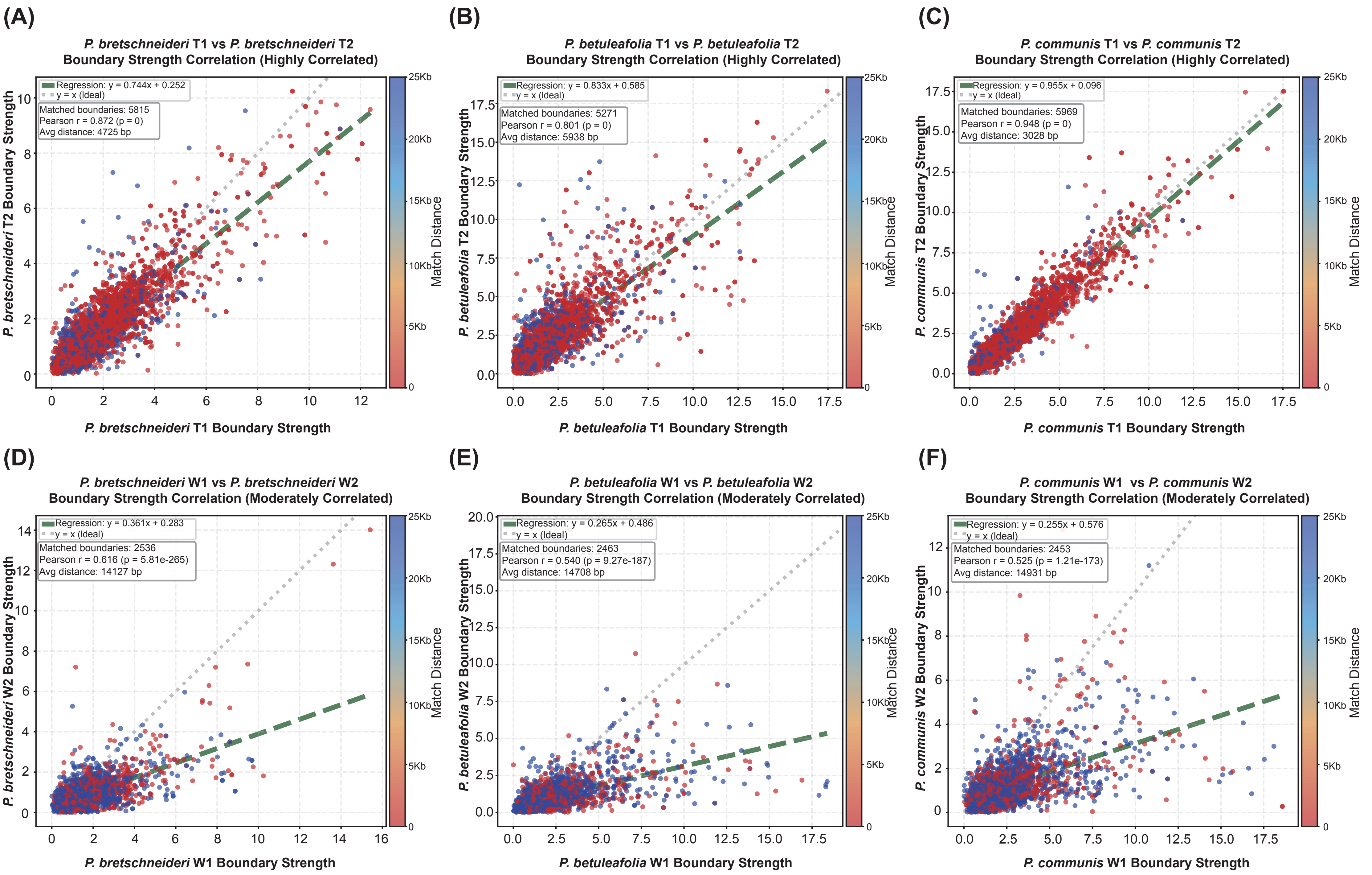
(A)
P. bretschneideri T1 vs P. bretschneideri T2
Boundary Strength Correlation (Highly Correlated)
Regression: y = 0.744x + 0.252
y = x (Ideal)
Matched boundaries: 5815
Pearson r = 0.872 (p = 0)
Avg distance: 4725 bp
P. bretschneideri T2 Boundary Strength
P. bretschneideri T1 Boundary Strength
Match Distance
(B)
P. betuleafolia T1 vs P. betuleafolia T2
Boundary Strength Correlation (Highly Correlated)
Regression: y = 0.833x + 0.585
y = x (Ideal)
Matched boundaries: 5271
Pearson r = 0.801 (p = 0)
Avg distance: 5938 bp
P. betuleafolia T2 Boundary Strength
P. betuleafolia T1 Boundary Strength
Match Distance
(C)
P. communis T1 vs P. communis T2
Boundary Strength Correlation (Highly Correlated)
Regression: y = 0.955x + 0.096
y = x (Ideal)
Matched boundaries: 5969
Pearson r = 0.948 (p = 0)
Avg distance: 3028 bp
P. communis T2 Boundary Strength
P. communis T1 Boundary Strength
Match Distance
(D)
P. bretschneideri W1 vs P. bretschneideri W2
Boundary Strength Correlation (Moderately Correlated)
Regression: y = 0.361x + 0.283
y = x (Ideal)
Matched boundaries: 2536
Pearson r = 0.616 (p = 5.81e-265)
Avg distance: 14127 bp
P. bretschneideri W2 Boundary Strength
P. bretschneideri W1 Boundary Strength
Match Distance
(E)
P. betuleafolia W1 vs P. betuleafolia W2
Boundary Strength Correlation (Moderately Correlated)
Regression: y = 0.265x + 0.486
y = x (Ideal)
Matched boundaries: 2463
Pearson r = 0.540 (p = 9.27e-187)
Avg distance: 14708 bp
P. betuleafolia W2 Boundary Strength
P. betuleafolia W1 Boundary Strength
Match Distance
(F)
P. communis W1 vs P. communis W2
Boundary Strength Correlation (Moderately Correlated)
Regression: y = 0.255x + 0.576
y = x (Ideal)
Matched boundaries: 2453
Pearson r = 0.525 (p = 1.21e-173)
Avg distance: 14931 bp
P. communis W2 Boundary Strength
P. communis W1 Boundary Strength
Match Distance
0
5Kb
10Kb
15Kb
20Kb
25Kb
