# Supplementary material for: 3D Chromatin Architecture Provides Insights Into Leaf Trait Variation Among Pear Species
Source: Adv Sci (Weinh). 2026 May 12;13(41):e19321. doi: 10.1002/advs.202519321 (PMC13335592; doi:10.1002/advs.202519321)
Supplement: Supplementary file 1 — Supporting File 1: advs75472‐sup‐0001‐FiguresS1‐S20.zip. [file ADVS-13-e19321-s002.zip › advs75472-sup-0014-FigureS14.pdf]

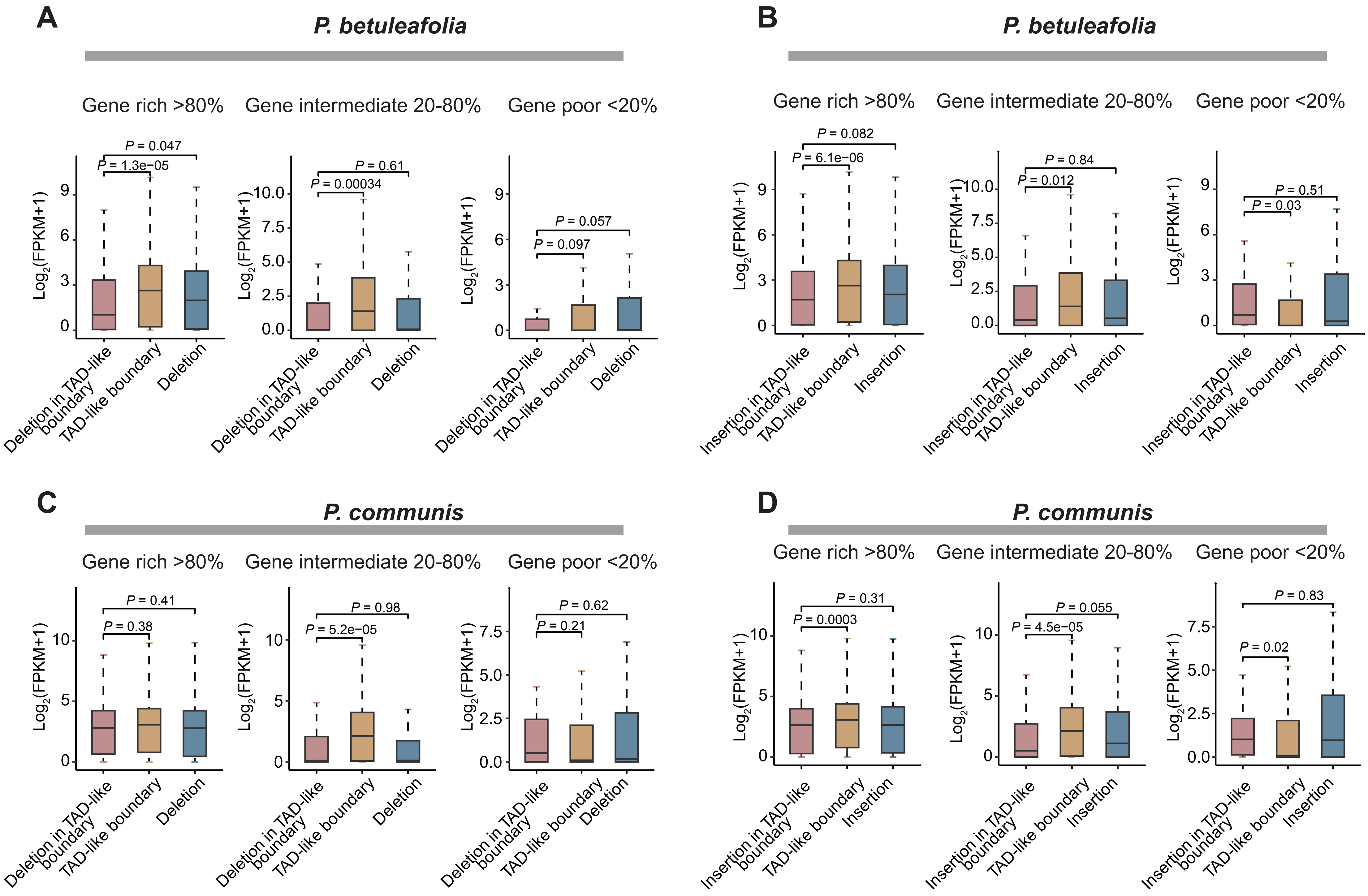

A
P. betuleafolia
Gene rich >80%
Gene intermediate 20-80%
Gene poor <20%
P = 0.047
P = 1.3e−05
P = 0.61
P = 0.00034
P = 0.057
P = 0.097
Log2(FPKM+1)
Deletion in TAD-like boundary
TAD-like boundary
Deletion
B
P. betuleafolia
Gene rich >80%
Gene intermediate 20-80%
Gene poor <20%
P = 0.082
P = 6.1e−06
P = 0.84
P = 0.012
P = 0.51
P = 0.03
Insertion in TAD-like boundary
TAD-like boundary
Insertion
C
P. communis
Gene rich >80%
Gene intermediate 20-80%
Gene poor <20%
P = 0.41
P = 0.38
P = 0.98
P = 5.2e−05
P = 0.62
P = 0.21
Deletion in TAD-like boundary
TAD-like boundary
Deletion
D
P. communis
Gene rich >80%
Gene intermediate 20-80%
Gene poor <20%
P = 0.31
P = 0.0003
P = 0.055
P = 4.5e−05
P = 0.83
P = 0.02
Insertion in TAD-like boundary
TAD-like boundary
Insertion
