# Supplementary figures and images for: 3D Chromatin Architecture Provides Insights Into Leaf Trait Variation Among Pear Species
Source: Adv Sci (Weinh). 2026 May 12;13(41):e19321. doi: 10.1002/advs.202519321 (PMC13335592; doi:10.1002/advs.202519321)

25kb

N=43 (filtered) 173 (unique) 173 (total), P2LL = 4.583

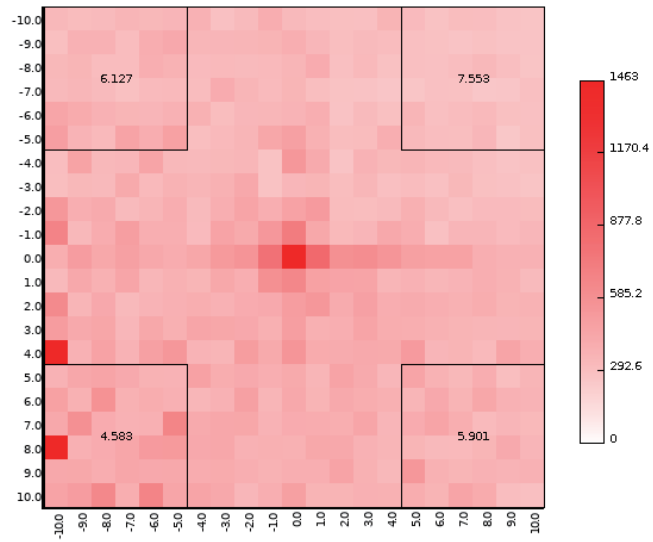

10kb

N=38 (filtered) 469 (unique) 469 (total), P2LL = 4.343

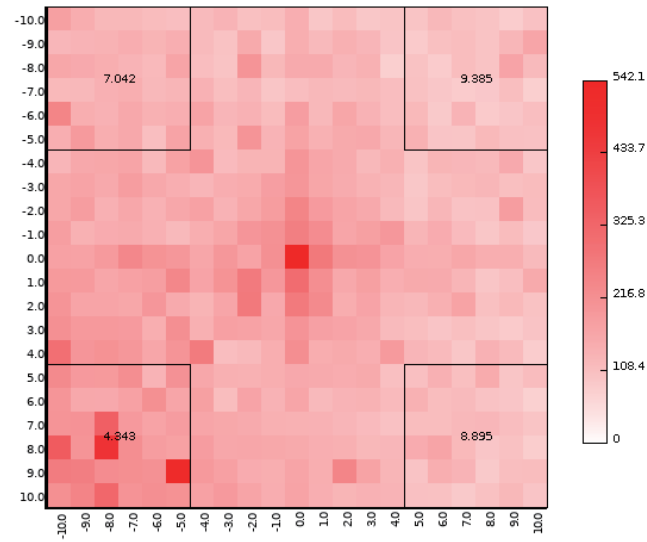

5kb

N=33 (filtered) 454 (unique) 454 (total), P2LL = 7.895

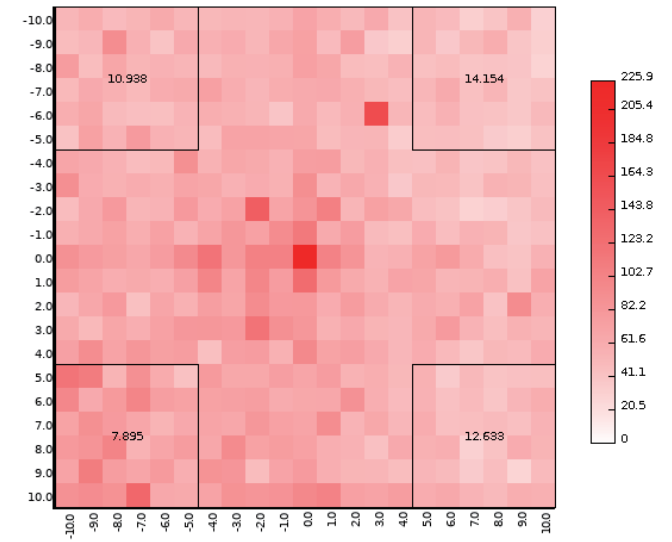

Supplement: Supplementary file 1 — Supporting File 1: advs75472‐sup‐0001‐FiguresS1‐S20.zip. [file ADVS-13-e19321-s002.zip › advs75472-sup-0002-FigureS2.pdf]

(a)

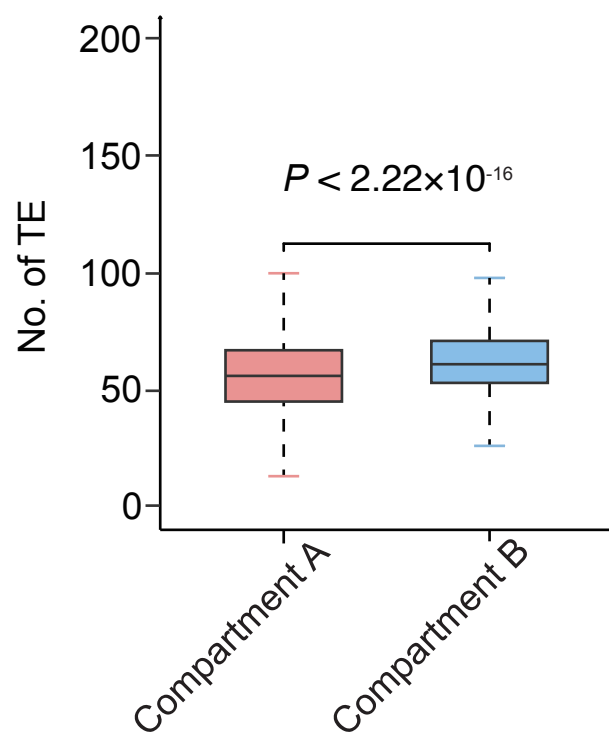

(b)

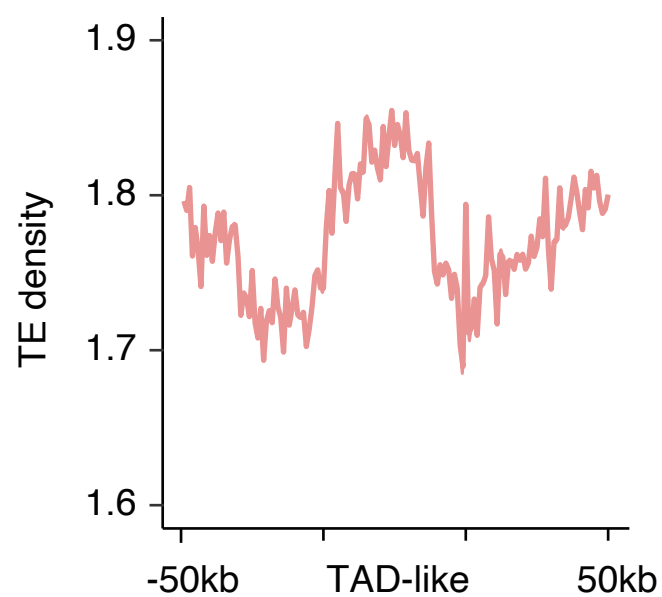

(c)

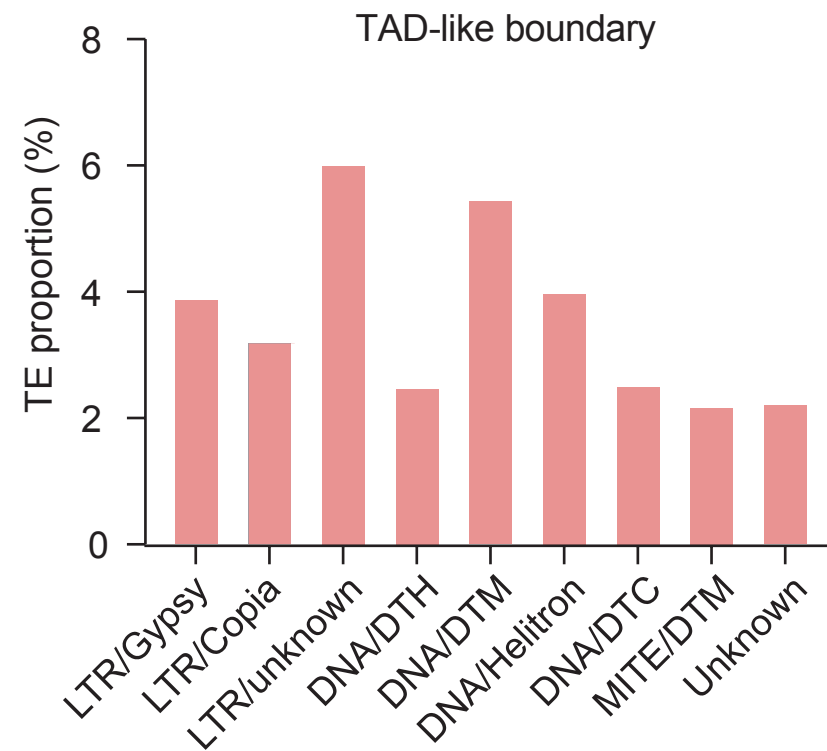

Supplement: Supplementary file 1 — Supporting File 1: advs75472‐sup‐0001‐FiguresS1‐S20.zip. [file ADVS-13-e19321-s002.zip › advs75472-sup-0003-FigureS3.pdf]

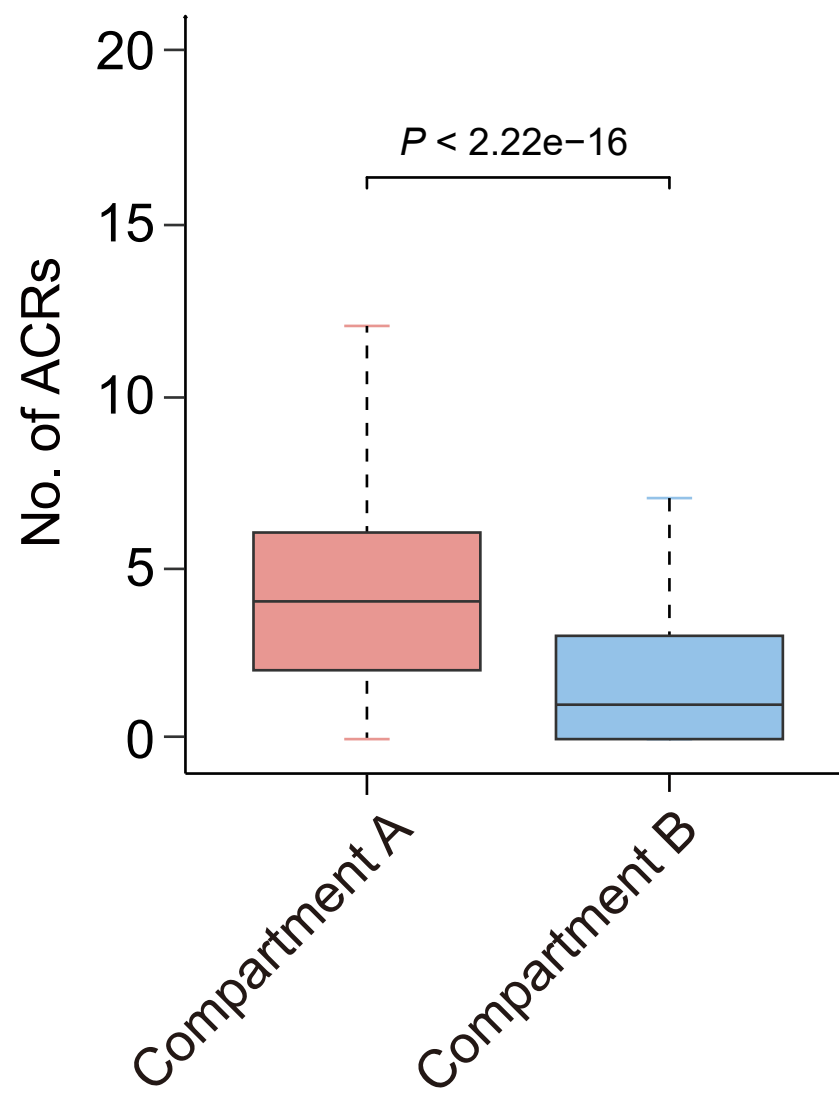

Supplement: Supplementary file 1 — Supporting File 1: advs75472‐sup‐0001‐FiguresS1‐S20.zip. [file ADVS-13-e19321-s002.zip › advs75472-sup-0004-FigureS4.pdf]

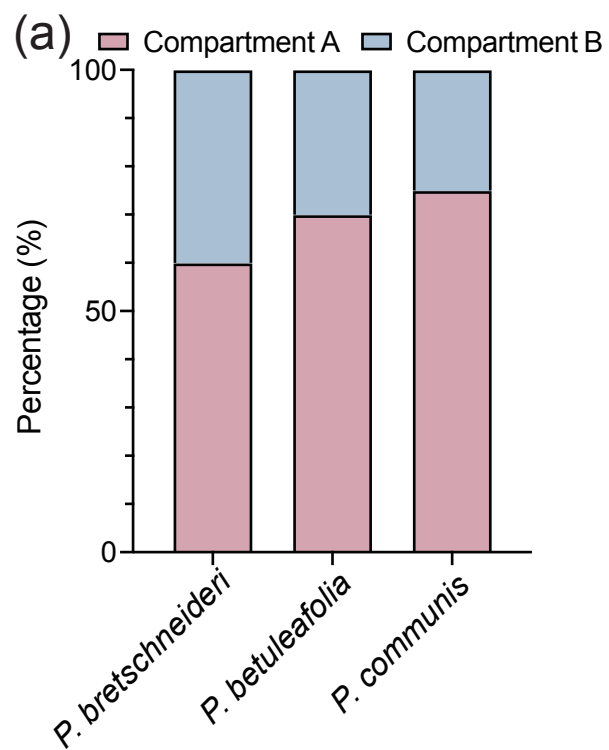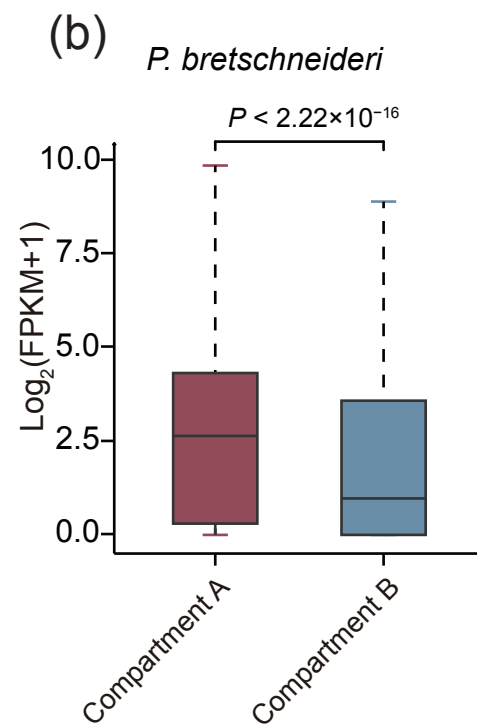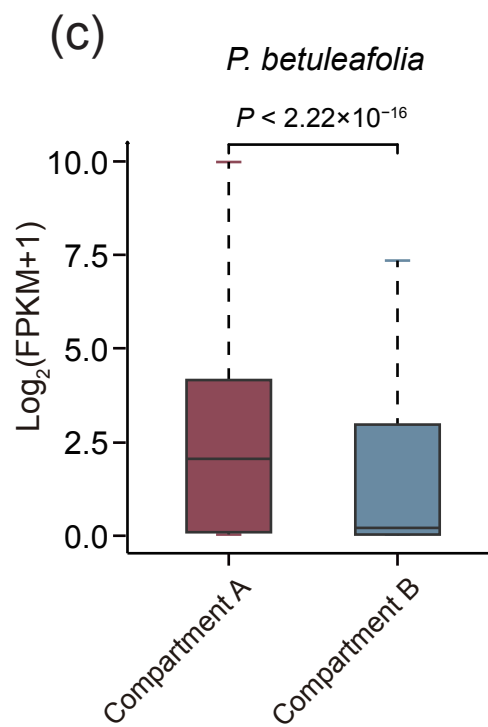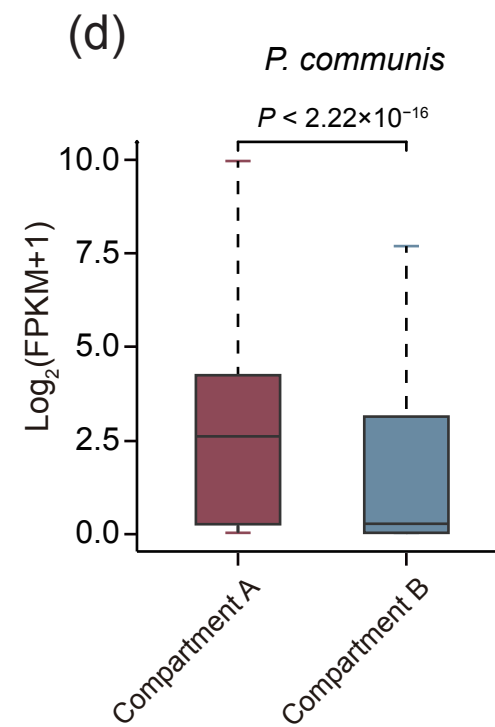

Supplement: Supplementary file 1 — Supporting File 1: advs75472‐sup‐0001‐FiguresS1‐S20.zip. [file ADVS-13-e19321-s002.zip › advs75472-sup-0005-FigureS5.pdf]

*P. betuleafolia* vs *P. bretschnideri*

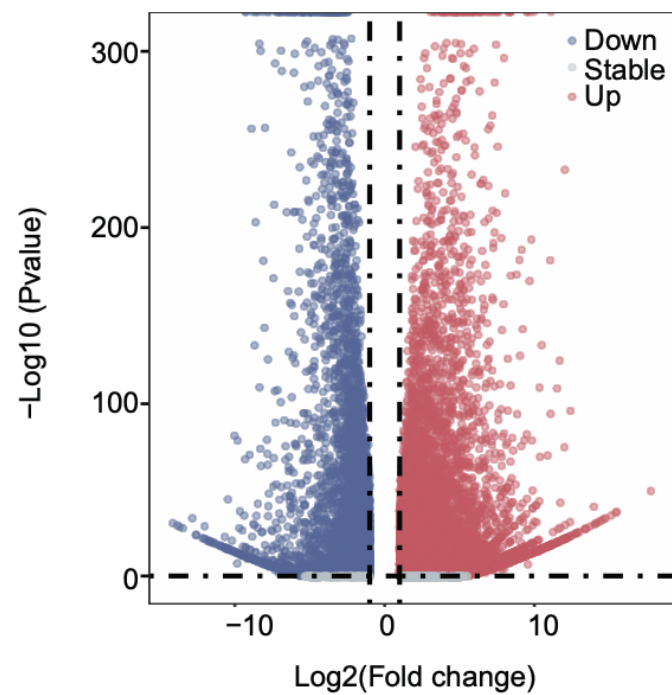

*P. bretschnideri* vs *P. communis*

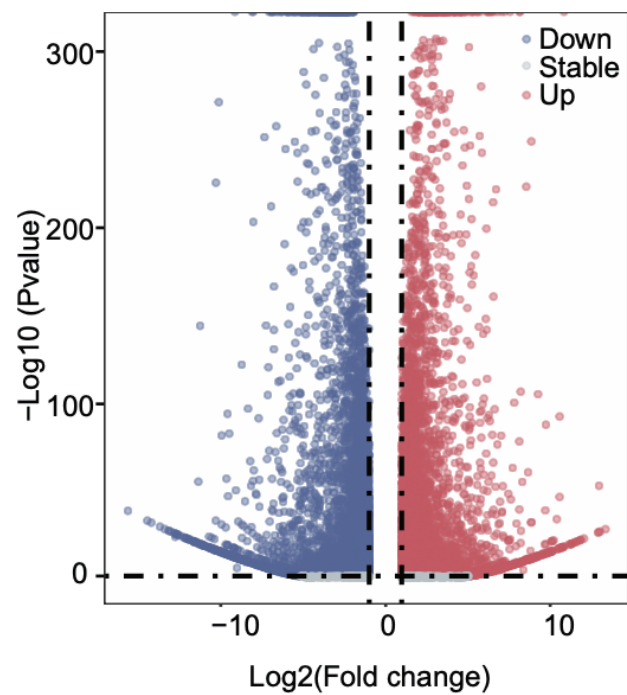

*P. betuleafolia* vs *P. communis*

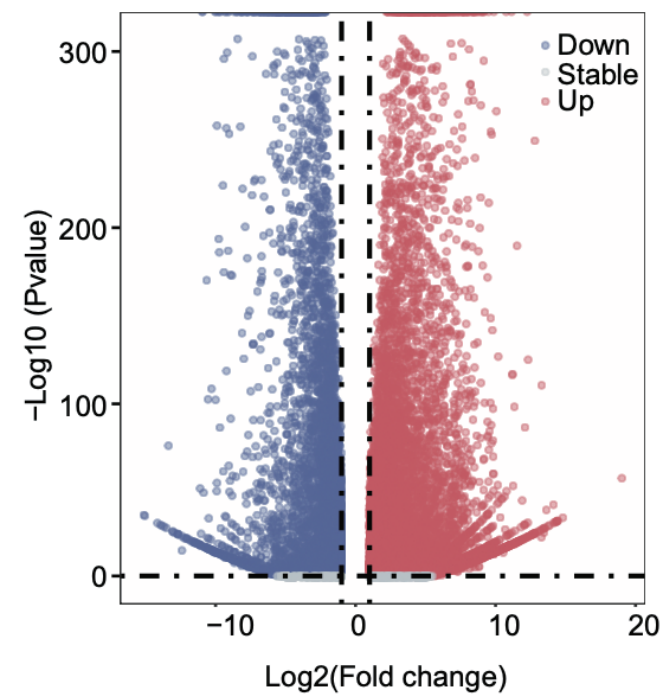

Supplement: Supplementary file 1 — Supporting File 1: advs75472‐sup‐0001‐FiguresS1‐S20.zip. [file ADVS-13-e19321-s002.zip › advs75472-sup-0006-FigureS6.pdf]

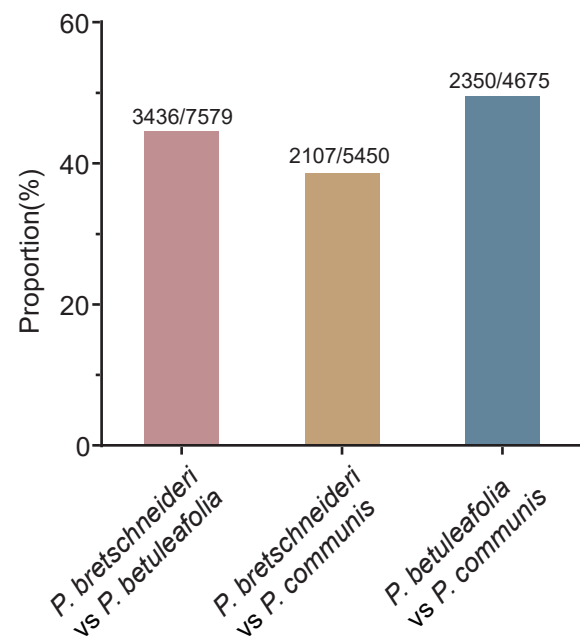

Supplement: Supplementary file 1 — Supporting File 1: advs75472‐sup‐0001‐FiguresS1‐S20.zip. [file ADVS-13-e19321-s002.zip › advs75472-sup-0007-FigureS7.pdf]

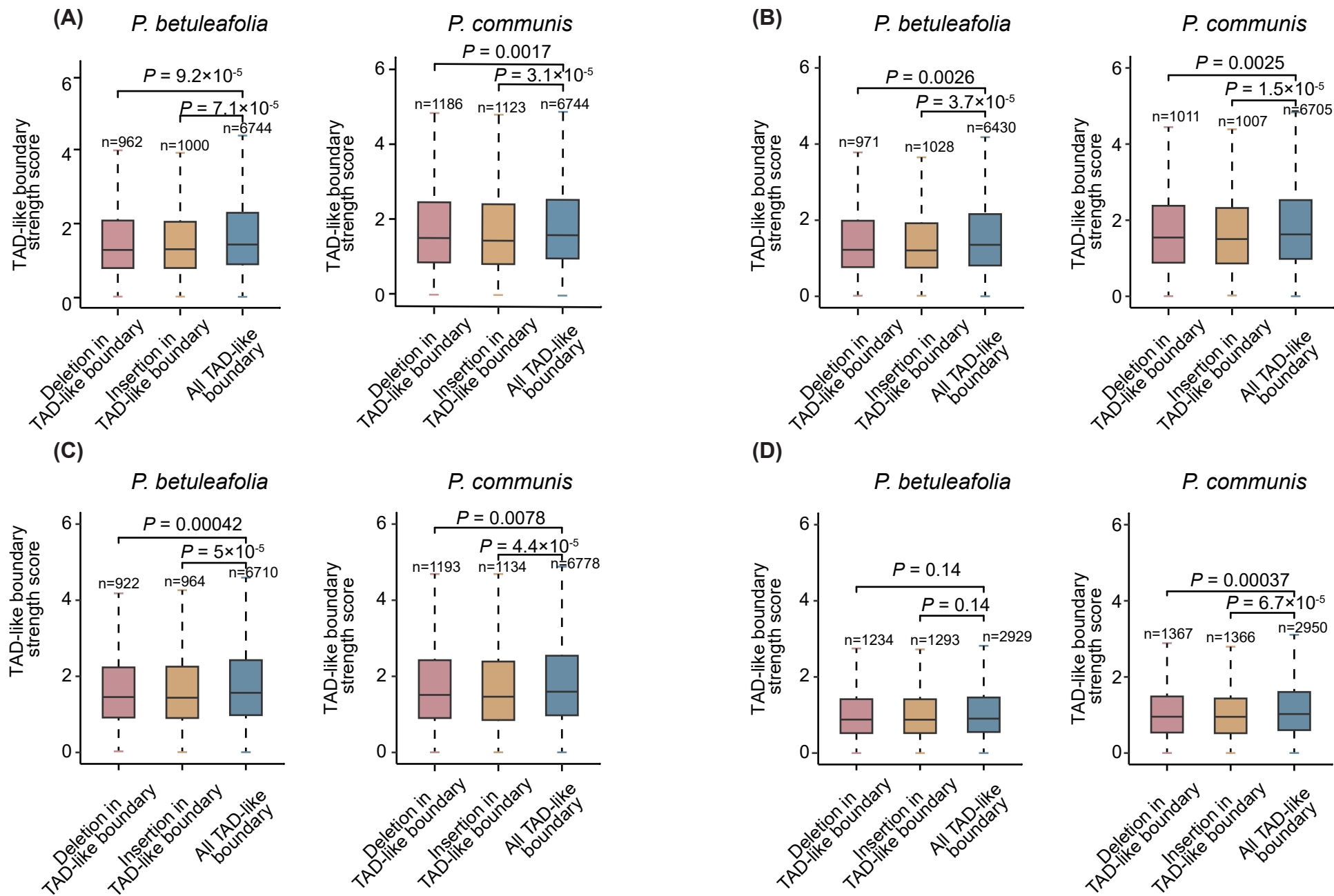

Supplement: Supplementary file 1 — Supporting File 1: advs75472‐sup‐0001‐FiguresS1‐S20.zip. [file ADVS-13-e19321-s002.zip › advs75472-sup-0012-FigureS12.pdf]

**A**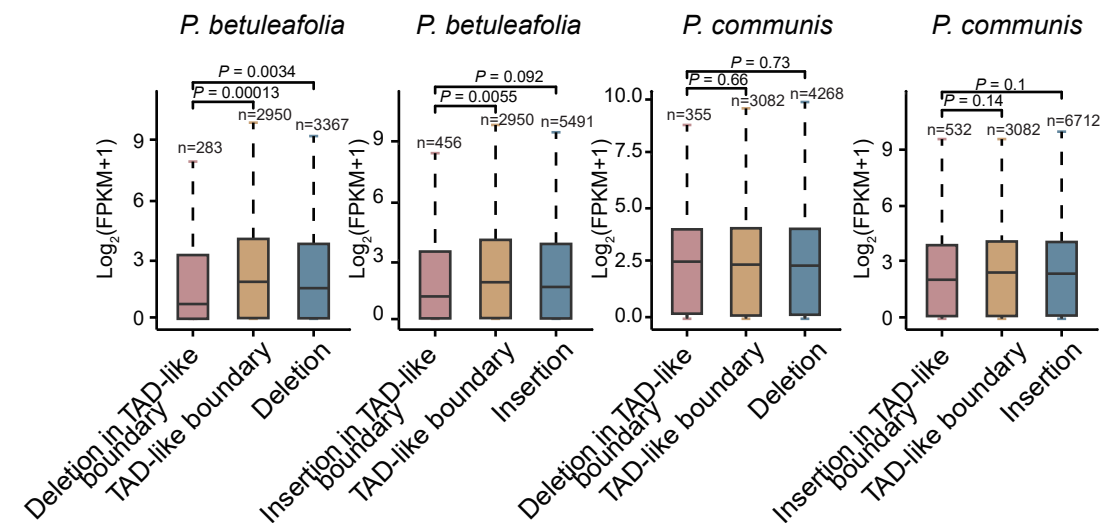**B**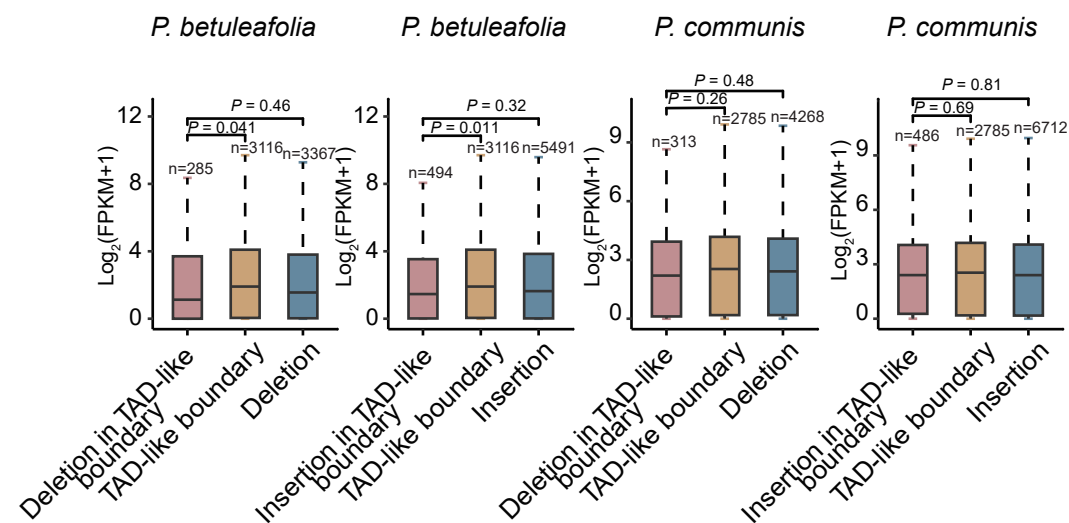**C**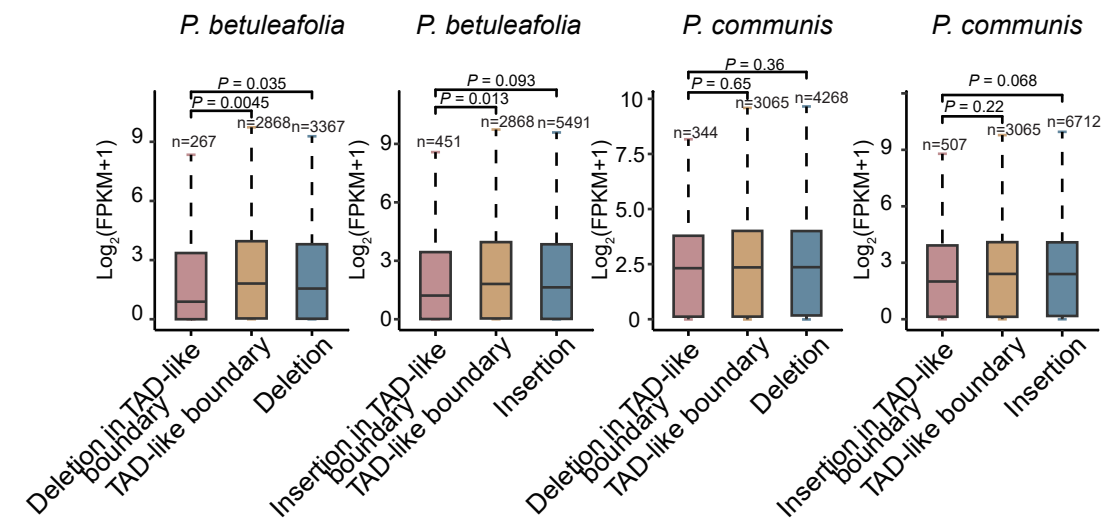**D**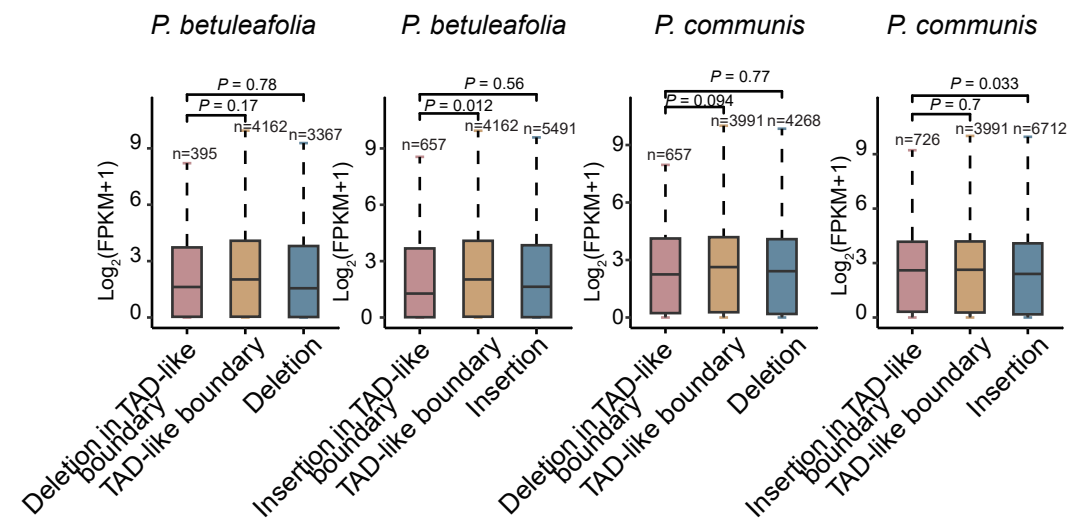

Supplement: Supplementary file 1 — Supporting File 1: advs75472‐sup‐0001‐FiguresS1‐S20.zip. [file ADVS-13-e19321-s002.zip › advs75472-sup-0013-FigureS13.pdf]

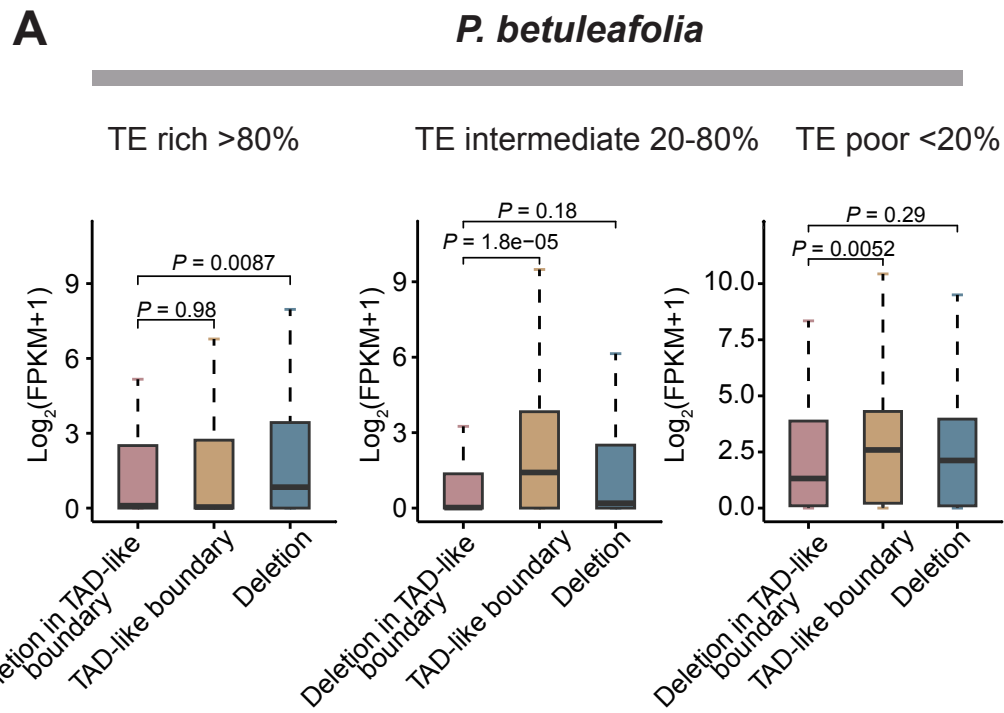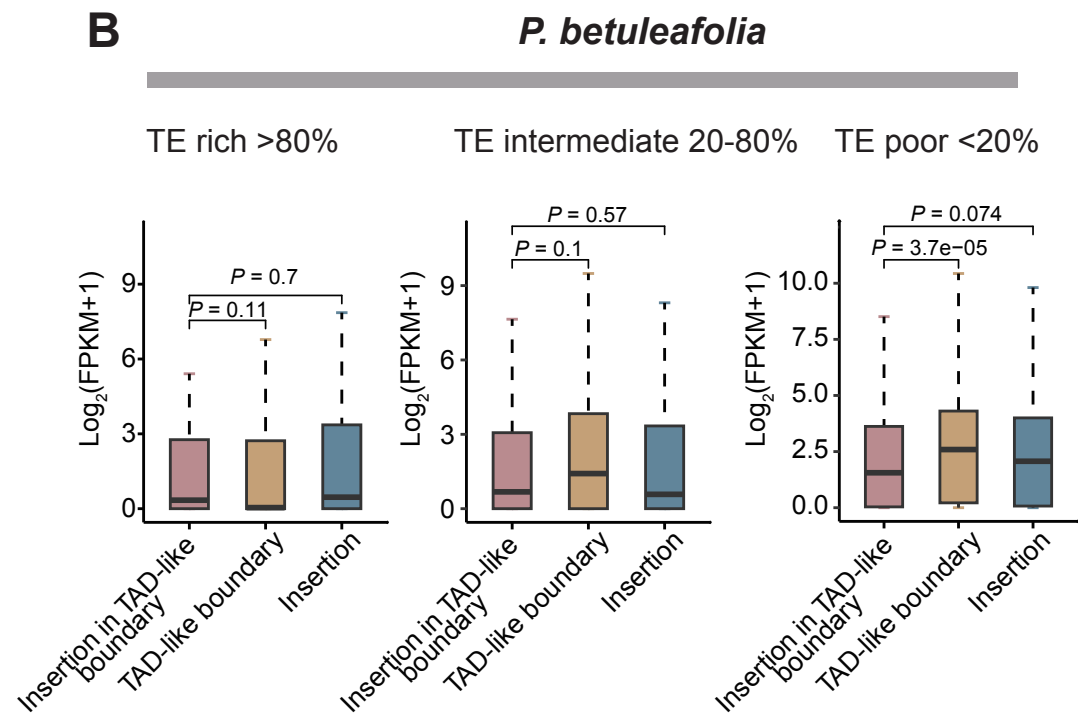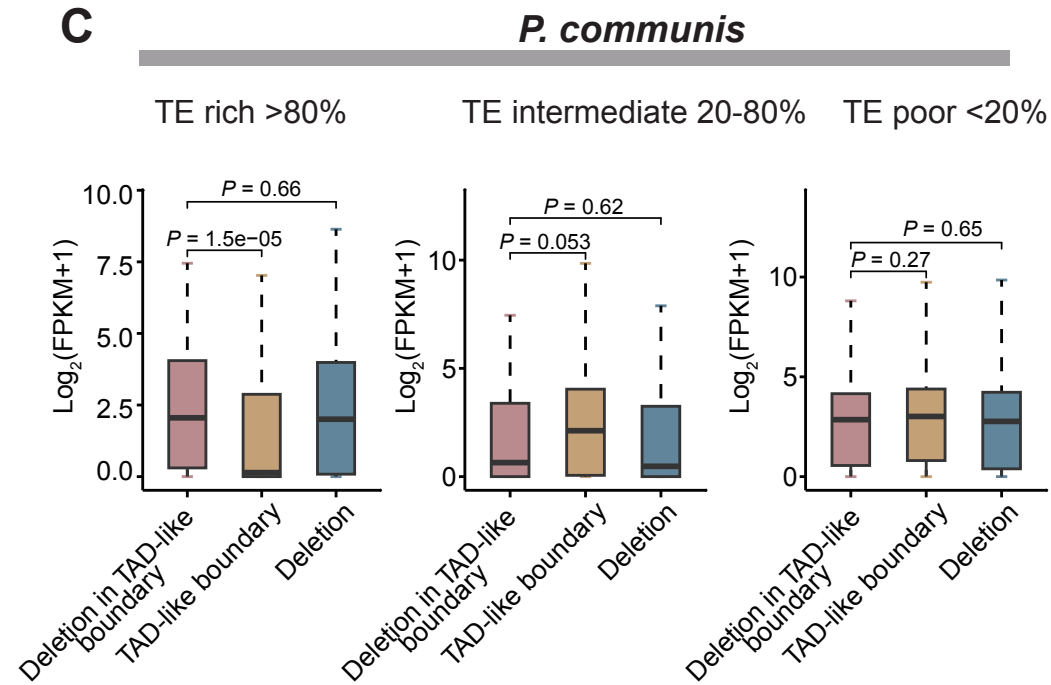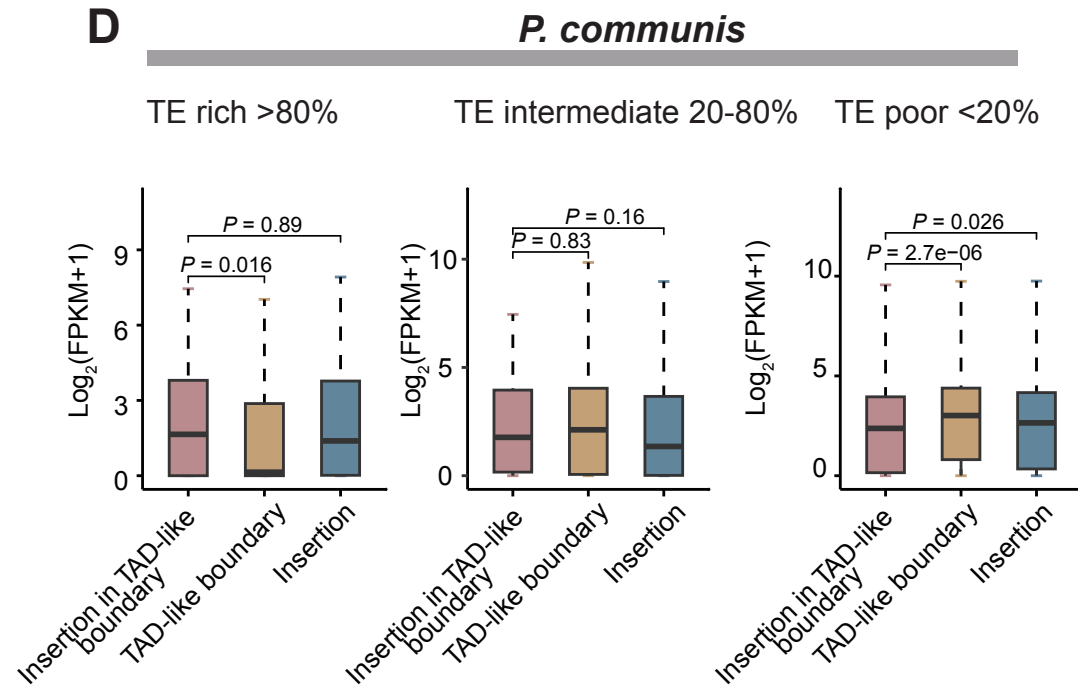

Supplement: Supplementary file 1 — Supporting File 1: advs75472‐sup‐0001‐FiguresS1‐S20.zip. [file ADVS-13-e19321-s002.zip › advs75472-sup-0015-FigureS15.pdf]

**A**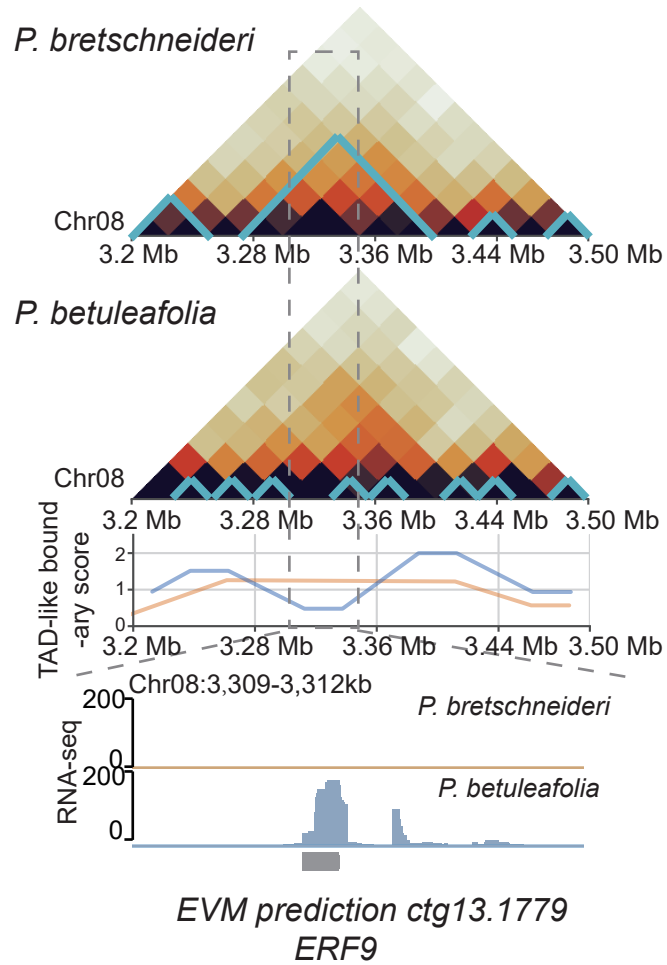**B**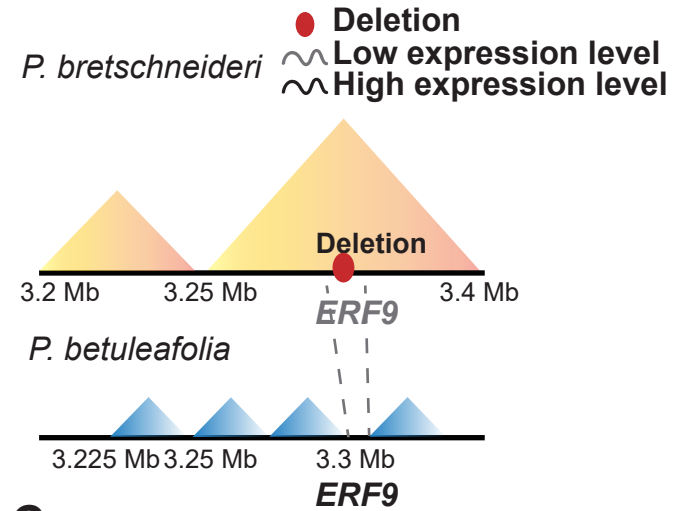**C**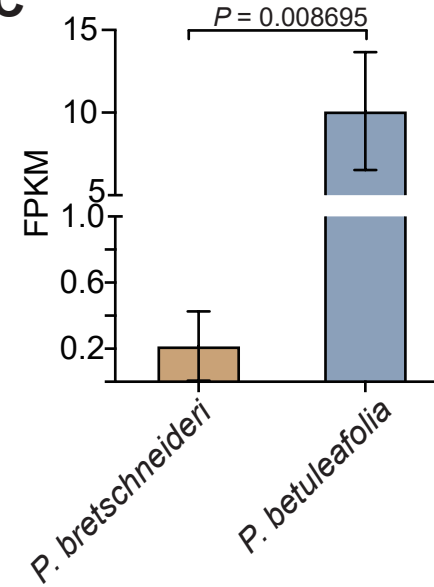

Supplement: Supplementary file 1 — Supporting File 1: advs75472‐sup‐0001‐FiguresS1‐S20.zip. [file ADVS-13-e19321-s002.zip › advs75472-sup-0016-FigureS16.pdf]

**A**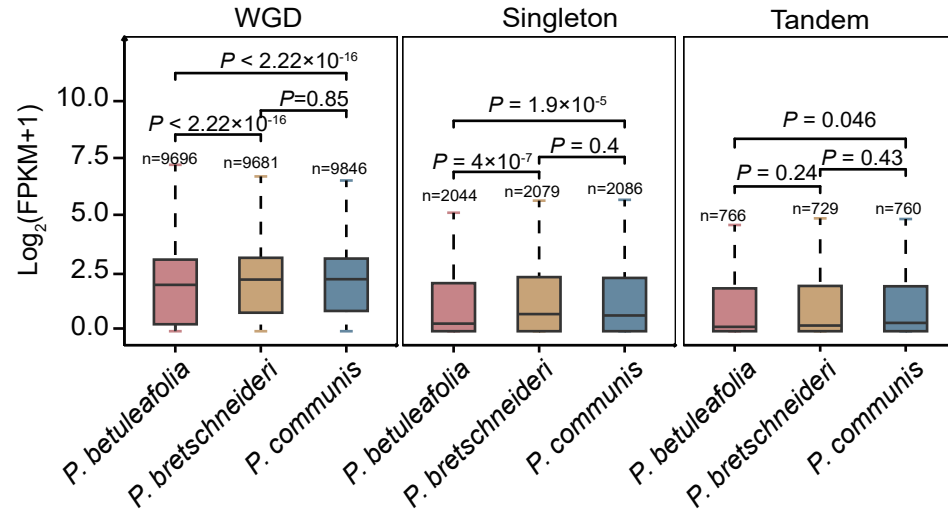**B**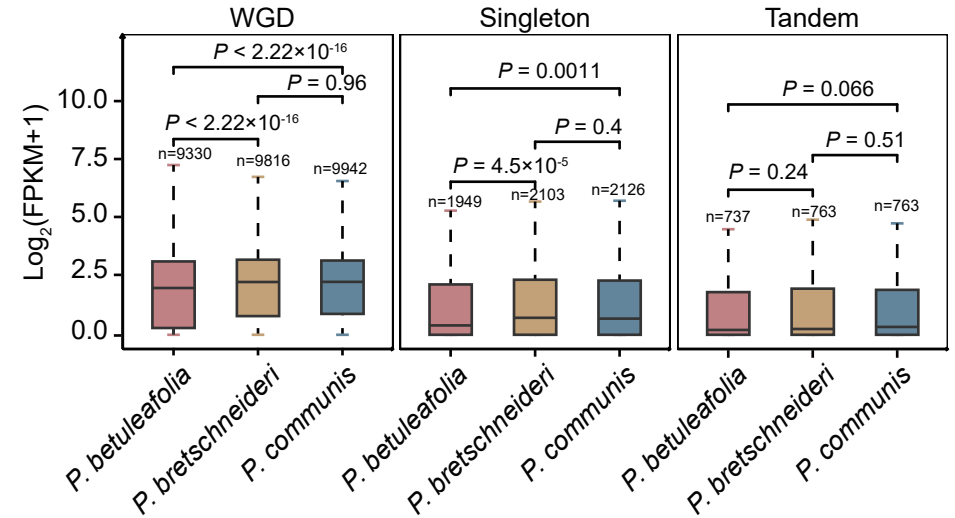**C**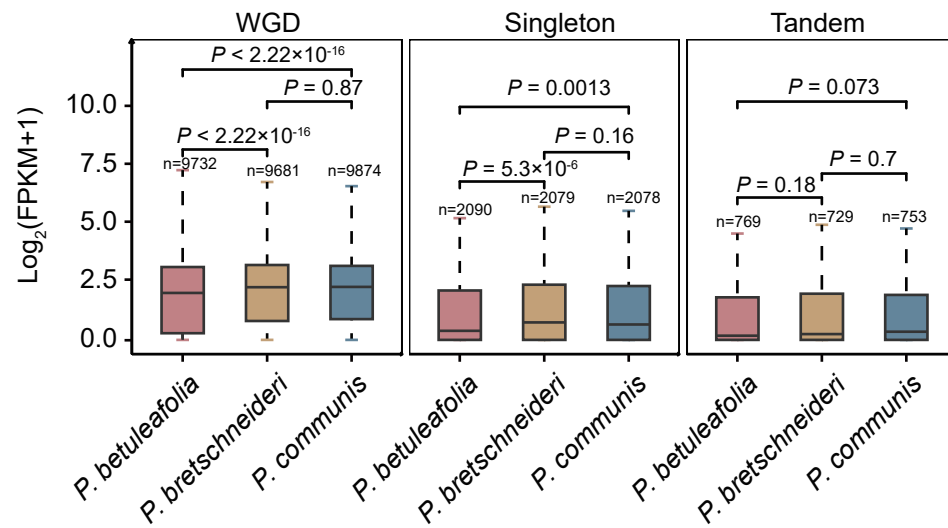**D**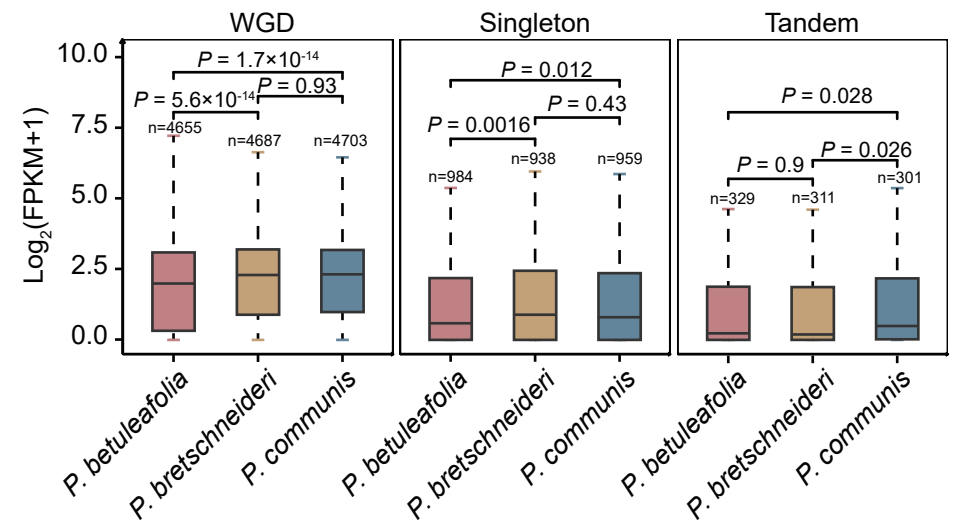

Supplement: Supplementary file 1 — Supporting File 1: advs75472‐sup‐0001‐FiguresS1‐S20.zip. [file ADVS-13-e19321-s002.zip › advs75472-sup-0017-FigureS17.pdf]

(a)

### Domestication

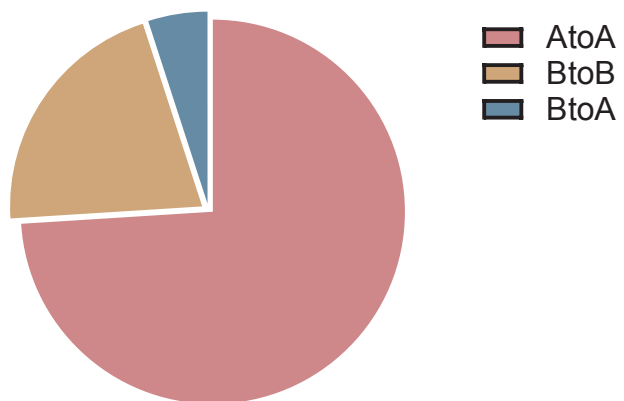

(b)

### Divergence

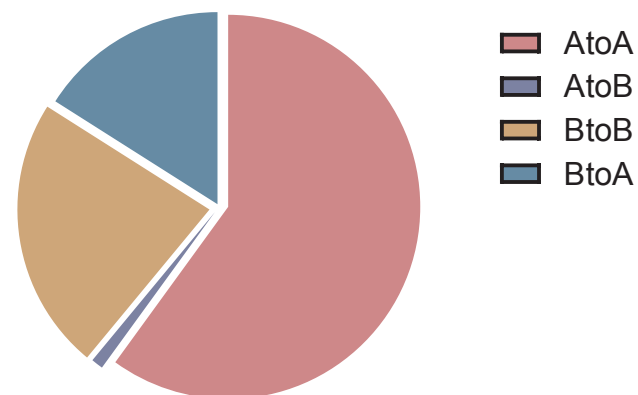

(c)

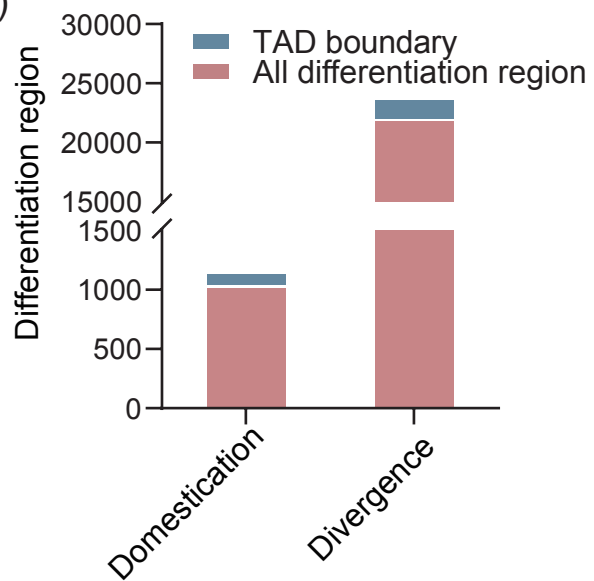

(d)

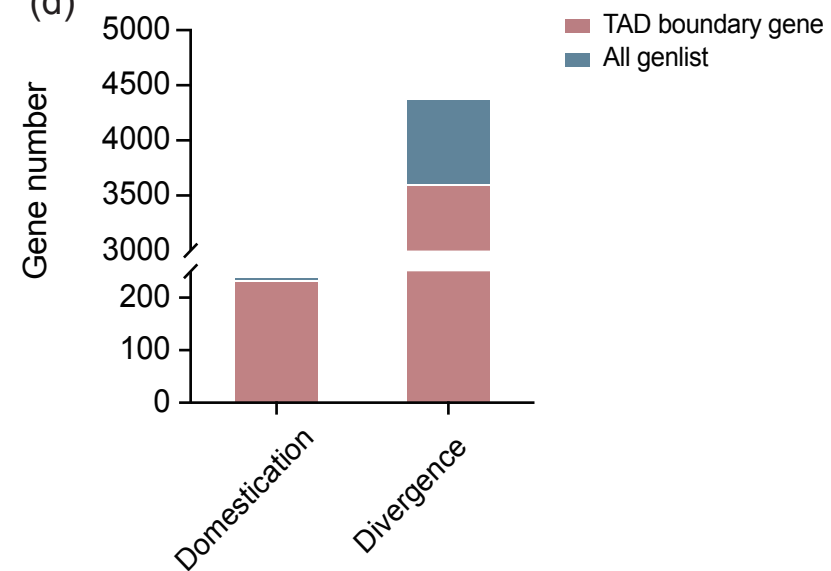

Supplement: Supplementary file 1 — Supporting File 1: advs75472‐sup‐0001‐FiguresS1‐S20.zip. [file ADVS-13-e19321-s002.zip › advs75472-sup-0018-FigureS18.pdf]

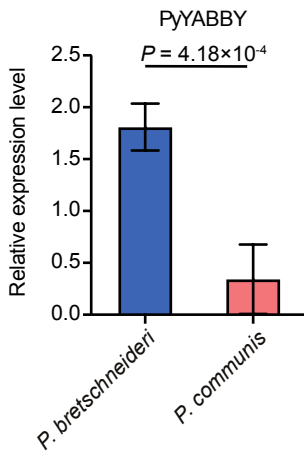

Supplement: Supplementary file 1 — Supporting File 1: advs75472‐sup‐0001‐FiguresS1‐S20.zip. [file ADVS-13-e19321-s002.zip › advs75472-sup-0019-FigureS19.pdf]

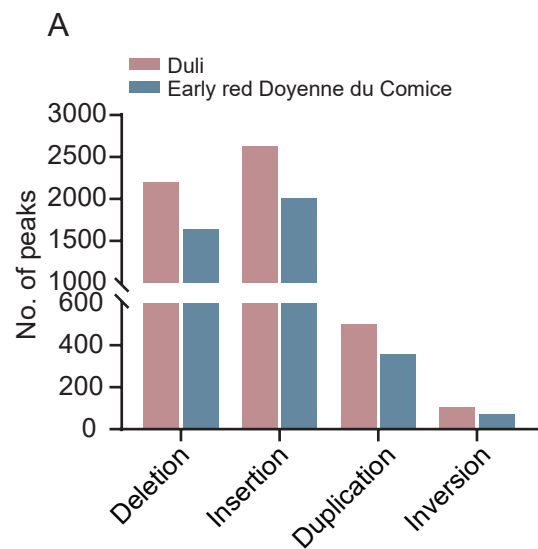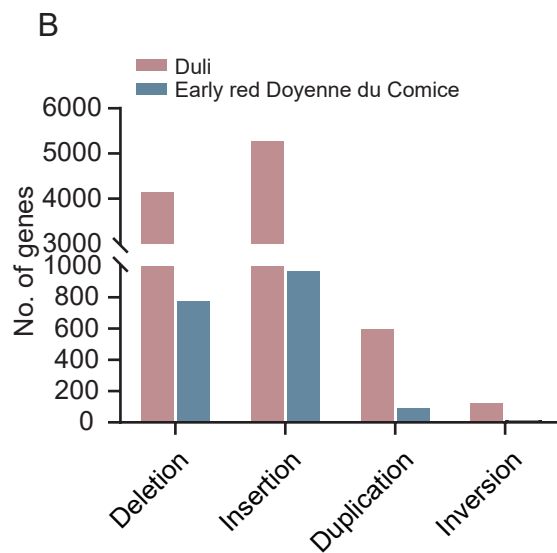

Supplement: Supplementary file 1 — Supporting File 1: advs75472‐sup‐0001‐FiguresS1‐S20.zip. [file ADVS-13-e19321-s002.zip › advs75472-sup-0020-Figure S20.pdf]

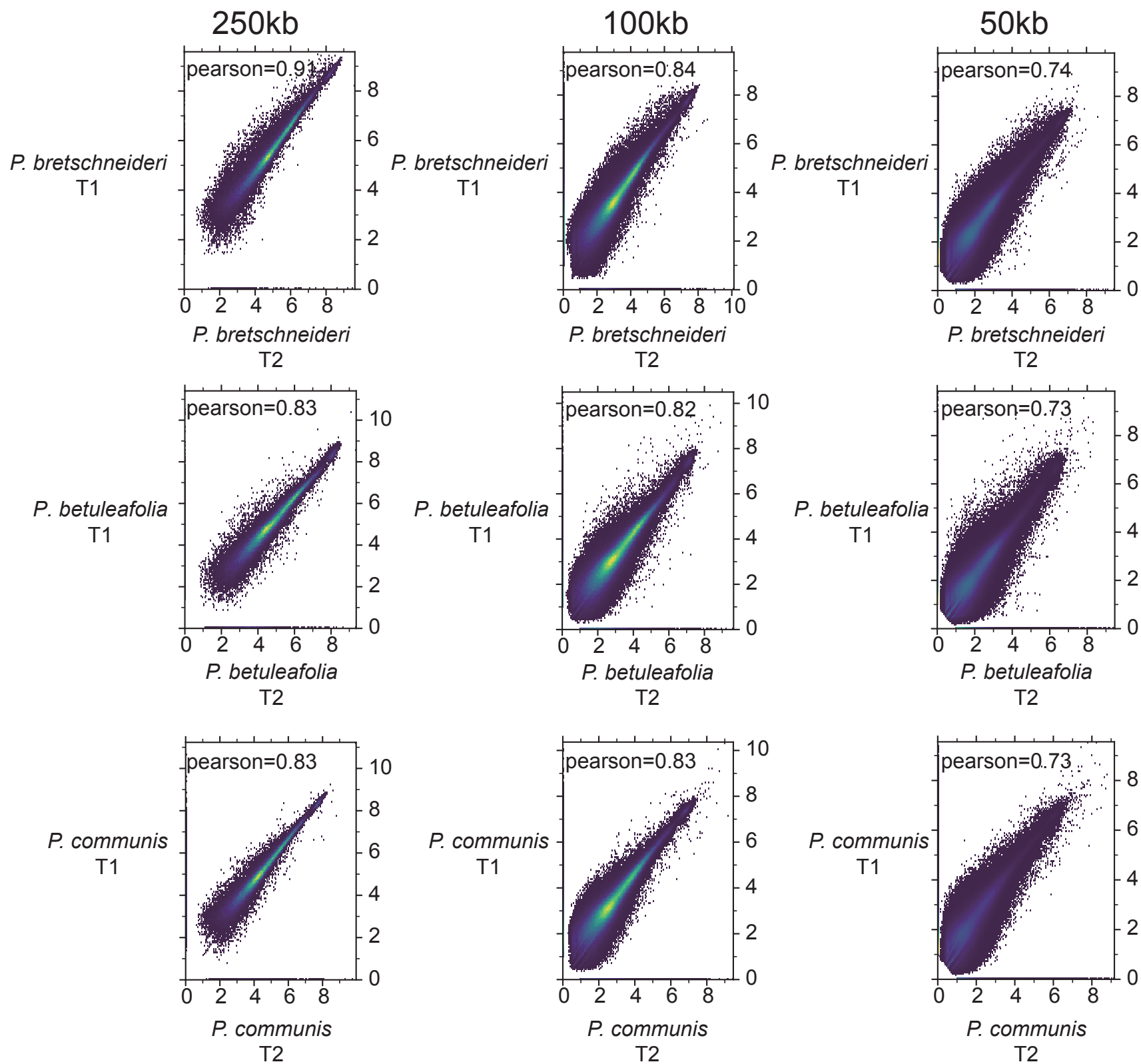

Supplement: Supplementary file 1 — Supporting File 1: advs75472‐sup‐0001‐FiguresS1‐S20.zip. [file ADVS-13-e19321-s002.zip › advs75472-sup-0001-FigureS1.pdf]
