# Supplementary material for: 3D Chromatin Architecture Provides Insights Into Leaf Trait Variation Among Pear Species
Source: Adv Sci (Weinh). 2026 May 12;13(41):e19321. doi: 10.1002/advs.202519321 (PMC13335592; doi:10.1002/advs.202519321)
Supplement: Supplementary file 2 — Supporting File 2: advs75472‐sup‐0002‐TablesS1‐S7.zip. [Correction added on 13 May 2026 after first online publication: supporting information file 2 is updated.] [file ADVS-13-e19321-s001.zip › advs75472-sup-0002-tabless1-s7/advs75472-sup-0021-TableS1.docx]

Table S1: The summary of transposons in ‘Dangshansuli’ hifi genome.

|  | Class | Count | bpMasked | %masked |
| --- | --- | --- | --- | --- |
| LTR | Copia | 42657 | 35306558 | 7.04% |
|  | Gypsy | 59301 | 64697056 | 12.90% |
|  | unknown | 88650 | 58974530 | 11.76% |
| TIR | CACTA | 34548 | 11428504 | 2.28% |
|  | Mutator | 106645 | 24614513 | 4.91% |
|  | PIF_Harbinger | 42871 | 14834739 | 2.96% |
|  | Tc1_Mariner | 3552 | 700278 | 0.14% |
|  | hAT | 24187 | 6915457 | 1.38% |
| nonLTR | LINE_element | 2104 | 1032114 | 0.21% |
|  | unknown | 140 | 86283 | 0.02% |
| nonTIR | helitron | 55244 | 14634097 | 2.92% |
| repeat_region |  | 35082 | 8461236 | 1.69% |
| total | interspersed | 494981 | 241685365 | 48.20% |
