# Supplementary material for: 3D Chromatin Architecture Provides Insights Into Leaf Trait Variation Among Pear Species
Source: Adv Sci (Weinh). 2026 May 12;13(41):e19321. doi: 10.1002/advs.202519321 (PMC13335592; doi:10.1002/advs.202519321)
Supplement: Supplementary file 2 — Supporting File 2: advs75472‐sup‐0002‐TablesS1‐S7.zip. [Correction added on 13 May 2026 after first online publication: supporting information file 2 is updated.] [file ADVS-13-e19321-s001.zip › advs75472-sup-0002-tabless1-s7/advs75472-sup-0022-TableS2.docx]

Table S2. Summary of gene function annotation.

| Type | Gene number | Percent (%) |
| --- | --- | --- |
| Swissprot | 27,135 | 66.11 |
| Orthologs | 37,687 | 91.81 |
| NR | 39,237 | 95.59 |
| Pfam | 32,563 | 79.33 |
| KEGG | 17,150 | 41.78% |
| GO | 18,717 | 45.60 |
| Annotated | 40,064 | 97.60 |
| Unannotated | 984 | 2.4 |
| Total | 41,048 | - |
