# Supplementary material for: 3D Chromatin Architecture Provides Insights Into Leaf Trait Variation Among Pear Species
Source: Adv Sci (Weinh). 2026 May 12;13(41):e19321. doi: 10.1002/advs.202519321 (PMC13335592; doi:10.1002/advs.202519321)
Supplement: Supplementary file 2 — Supporting File 2: advs75472‐sup‐0002‐TablesS1‐S7.zip. [Correction added on 13 May 2026 after first online publication: supporting information file 2 is updated.] [file ADVS-13-e19321-s001.zip › advs75472-sup-0002-tabless1-s7/advs75472-sup-0023-TableS3.docx]

Table S3. Hi-C data statistics for three pear species.

|  | *P. bretschneideri* | *P. betuleafolia* | *P. communis* |
| --- | --- | --- | --- |
| Sequenced Read Pairs | 674,009,959 | 674,009,959 | 674,009,959 |
| Unique Reads | 476,889,199 | 437,227,204 | 424,109,243 |
| Hi-C Contacts | 280,855,892 | 220,878,374 | 203,395,483 |
| Inter-chromosomal | 90,647,950 | 65,485,299 | 63,663,539 |
| Intra-chromosomal | 190,207,942 | 155,393,075 | 139,731,944 |
| Short Range (<20Kb) | 80,250,133 | 74,624,627 | 68,103,522 |
| Long Range (>20Kb) | 109,956,714 | 80,767,691 | 71,627,179 |
