# Supplementary material for: 3D Chromatin Architecture Provides Insights Into Leaf Trait Variation Among Pear Species
Source: Adv Sci (Weinh). 2026 May 12;13(41):e19321. doi: 10.1002/advs.202519321 (PMC13335592; doi:10.1002/advs.202519321)
Supplement: Supplementary file 2 — Supporting File 2: advs75472‐sup‐0002‐TablesS1‐S7.zip. [Correction added on 13 May 2026 after first online publication: supporting information file 2 is updated.] [file ADVS-13-e19321-s001.zip › advs75472-sup-0002-tabless1-s7/advs75472-sup-0025-TableS5.docx]

Supplemental Table S5. Structural variation (SV) distribution around TAD boundaries of *P. betuleafolia* and *P. communis*

|  | ABA | | PBA | | NBA | |
| --- | --- | --- | --- | --- | --- | --- |
| SV number | *P. betuleafolia* | *P. communis* | *P. betuleafolia* | *P. communis* | *P. betuleafolia* | *P. communis* |
| Deletion | 9 | 2 | 2736 | 3671 | 4238 | 5394 |
| Insertion | 0 | 0 | 2899 | 3669 | 4471 | 5378 |
| Duplication | 11 | 3 | 102 | 47 | 104 | 50 |
| Inversion | 0 | 2 | 36 | 43 | 41 | 51 |

Note: ABA SVs were defined as SVs spanning the whole length of one boundary, PBA SVs were defined as SVs spanning part of the length of one boundary, and NBA SVs were defined as SVs within TADs
