# Supplementary material for: 3D Chromatin Architecture Provides Insights Into Leaf Trait Variation Among Pear Species
Source: Adv Sci (Weinh). 2026 May 12;13(41):e19321. doi: 10.1002/advs.202519321 (PMC13335592; doi:10.1002/advs.202519321)
Supplement: Supplementary file 2 — Supporting File 2: advs75472‐sup‐0002‐TablesS1‐S7.zip. [Correction added on 13 May 2026 after first online publication: supporting information file 2 is updated.] [file ADVS-13-e19321-s001.zip › advs75472-sup-0002-tabless1-s7/advs75472-sup-0026-TableS6.docx]

Table S6. List of 65 pear germplasm accessions

| **Sample Name** | **Species** | **Origin** |  | **Group** |
| --- | --- | --- | --- | --- |
|  |  |  | **Cultivated** |  |
|  |  |  | **or wild** |  |
|  |  |  |  |  |
| Pyc_br1 | *P. bretschneideri* | China | Cultivated | Asian |
| Pyc_br2 | *P. bretschneider*i | China | Cultivated | Asian |
| Pyc_br3 | *P. bretschneideri* | China | Cultivated | Asian |
| Pyc_br4 | *P. bretschneider*i | China | Cultivated | Asian |
| Pyc_br5 | *P. bretschneideri* | China | Cultivated | Asian |
| Pyc_py1 | *P. pyrifolia* | China | Cultivated | Asian |
| Pyc_py2 | *P. pyrifolia* | Korea | Cultivated | Asian |
| Pyc_py3 | P*. pyrifolia* | Japan | Cultivated | Asian |
| Pyc_py4 | *P. pyrifolia* | China | Cultivated | Asian |
| Pyc_py5 | *P. pyrifolia* | China | Cultivated | Asian |
| Pyc_py6 | *P. pyrifolia* | China | Cultivated | Asian |
| Pyc_py7 | *P. pyrifolia* | China | Cultivated | Asian |
| Pyc_py8 | *P. pyrifolia* | Japan | Cultivated | Asian |
| Pyc_py9 | *P. pyrifolia* | Japan | Cultivated | Asian |
| Pyc_py10 | *P. pyrifolia* | Japan | Cultivated | Asian |
| Pyc_py11 | *P. pyrifolia* | Japan | Cultivated | Asian |
| Pyc_py12 | *P. pyrifolia* | Japan | Cultivated | Asian |
| Pyc_py13 | *P. pyrifolia* | Japan | Cultivated | Asian |
| Pyc_py14 | *P. pyrifoli*a | China | Cultivated | Asian |
| Pyc_py15 | *P. pyrifolia* | Japan | Cultivated | Asian |
| Pyc_py16 | *P. pyrifolia* | China | Cultivated | Asian |
| Pyc_py17 | *P. pyrifolia* | China | Cultivated | Asian |
| Pyc_co1 | *P. communis* | England | Cultivated | European |
| Pyc_co2 | *P. communis* | France | Cultivated | European |
| Pyc_co3 | *P. communis* | France | Cultivated | European |
| Pyc_co4 | *P. communis* | England | Cultivated | European |
| Pyc_co5 | *P. communis* | America | Cultivated | European |
| Pyc_co8 | *P. communis* | France | Cultivated | European |
| Pyc_co9 | *P. communis* | France | Cultivated | European |
| Pyc_co10 | *P. communis* | Poland | Cultivated | European |
| Pyc_co11 | *P. communis* | France | Cultivated | European |
| Pyc_co12 | *P. communis* | Italy | Cultivated | European |
| Pyc_co13 | *P. communi*s | Italy | Cultivated | European |
| Pyc_co14 | *P. communis* | Czech | Cultivated | European |
| Pyc_co15 | *P. communis* | Poland | Cultivated | European |
| Pyc_co16 | *P. communis* | America | Cultivated | European |
| Pyc_co17 | *P. communis* | Bulgaria | Cultivated | European |
| Pyc_co18 | *P. communis* | German | Cultivated | European |
| Pyc_co19 | *P. communis* | Australia | Cultivated | European |
| Pyc_co20 | *P. communis* | Bulgaria | Cultivated | European |
| Pyc_co21 | *P. communis* | America | Cultivated | European |
| Pyc_co22 | *P. communis* | German | Cultivated | European |
| Pyc_co23 | *P. communis* | German | Cultivated | European |
| Pyc_co24 | *P.communi*s | Soviet Union | Cultivated | European |
| Pyc_co25 | *P. communis* | Czech | Cultivated | European |
| Pyc_co26 | *P. communis* | Italy | Cultivated | European |
| Pyw_be1 | *P. betuleafolia* | China | Wild | Asian |
| Pyw_be2 | *P. betuleafolia* | China | Wild | Asian |
| Pyw_be3 | *P. betuleafolia* | China | Wild | Asian |
| Pyw_ca1 | *P. calleryana* | China | Wild | Asian |
| Pyw_ca2 | *P. calleryana* | China | Wild | Asian |
| Pyw_ca3 | *P. dimorphophylla* | Japan | Wild | Asian |
| Pyw_fa1 | *P. fauriei* | Korea | Wild | Asian |
| Pyw_fa2 | *P. fauriei* | Korea | Wild | Asian |
| Pyw_ko2 | *P. koehnei* | China | Wild | Asian |
| Pyw_pa1 | *P. pashia* | China | Wild | Asian |
| Pyw_pa2 | *P. pashia* | China | Wild | Asian |
| Pyw_pa3 | *P. pashia* | China | Wild | Asian |
| Pyw_ps1 | *P. pseudopashia* | China | Wild | Asian |
| Pyw_ps2 | *P. pseudopashia* | China | Wild | Asian |
| Pyw_py1 | *P. pyrifolia* | China | Wild | Asian |
| Pyw_py2 | *P. pyrifolia* | China | Wild | Asian |
| Pyw_py3 | *P. hondoensis* | Japan | Wild | Asian |
| Pyw_se1 | *P. serrulata* | China | Wild | Asian |
| Pyw_se2 | *P. serrulata* | China | Wild | Asian |
