# Supplementary material for: 3D Chromatin Architecture Provides Insights Into Leaf Trait Variation Among Pear Species
Source: Adv Sci (Weinh). 2026 May 12;13(41):e19321. doi: 10.1002/advs.202519321 (PMC13335592; doi:10.1002/advs.202519321)
Supplement: Supplementary file 2 — Supporting File 2: advs75472‐sup‐0002‐TablesS1‐S7.zip. [Correction added on 13 May 2026 after first online publication: supporting information file 2 is updated.] [file ADVS-13-e19321-s001.zip › advs75472-sup-0002-tabless1-s7/advs75472-sup-0027-TableS7.docx]

Tables S7. qPCR primer sequences

| Primer ID | Sequence of primers (5’-3’) |  |
| --- | --- | --- |
| qpcr-YABBY-F | TGAACATTTTCCAACGCCCG | qPCR primer |
| qpcr-YABBY-R | AGTGAGCCCAATTCTTGGCA |  |
